# MRN1 Implicates Chromatin Remodeling Complexes and Architectural Factors in mRNA Maturation

**DOI:** 10.1371/journal.pone.0044373

**Published:** 2012-09-18

**Authors:** Louis Düring, Michael Thorsen, Darima Sophia Njama Petersen, Brian Køster, Torben Heick Jensen, Steen Holmberg

**Affiliations:** 1 Department of Biology, Copenhagen BioCenter, University of Copenhagen, Copenhagen, Denmark; 2 Centre for mRNP Biogenesis and Metabolism, Department of Molecular Biology, Aarhus University, Aarhus, Denmark; St. Georges University of London, United Kingdom

## Abstract

A functional relationship between chromatin structure and mRNA processing events has been suggested, however, so far only a few involved factors have been characterized. Here we show that *rsc nhp6ΔΔ* mutants, deficient for the function of the chromatin remodeling factor RSC and the chromatin architectural proteins Nhp6A/Nhp6B, accumulate intron-containing pre-mRNA at the restrictive temperature. In addition, we demonstrate that *rsc8-ts16 nhp6ΔΔ* cells contain low levels of U6 snRNA and U4/U6 di-snRNA that is further exacerbated after two hours growth at the restrictive temperature. This change in U6 snRNA and U4/U6 di-snRNA levels in *rsc8-ts16 nhp6ΔΔ* cells is indicative of splicing deficient conditions. We identify *MRN1* (multi-copy suppressor of *rsc nhp6ΔΔ*) as a growth suppressor of *rsc nhp6ΔΔ* synthetic sickness. Mrn1 is an RNA binding protein that localizes both to the nucleus and cytoplasm. Genetic interactions are observed between *2 µm-MRN1* and the splicing deficient mutants *snt309Δ*, *prp3*, *prp4*, and *prp22*, and additional genetic analyses link *MRN1*, *SNT309*, *NHP6A/B*, *SWI/SNF*, and *RSC* supporting the notion of a role of chromatin structure in mRNA processing.

## Introduction

In eukaryotes, DNA is packaged into chromatin, which can inhibit the accessibility of DNA binding factors to their cognate sites *in vivo*. Thus, chromatin structural changes play a central role in controlling gene transcription as the formation of transcripts must contend with the repressive chromatin [1]. For active transcription to take place nucleosomes, the basic units of chromatin, need to be remodeled. ATP-dependent remodelers containing a catalytic subunit belonging to the Swi2/Snf2 family of ATPases, induce conformational changes in nucleosomes by altering histone-DNA interaction. In the Swi2/Snf2 family four different subclasses of remodelers are recognized: SWI/SNF, ISWI, CHD and INO80, that are all conserved from yeast to metazoans [2]. The yeast *Saccharomyces cerevisiae* contains the founding family member, SWI/SNF, and the highly related RSC (remodels the structure of chromatin) complex. RSC is abundant and holds fifteen-subunits with central roles in transcription [3], [4], DNA repair [5] and chromosome segregation [6]. Moreover, a genome-wide location analysis indicated that RSC is recruited to both RNA polymerase II (RNAPII) and RNA polymerase III (RNAPIII) promoters [7] and recently it was shown that RSC regulates nucleosome positioning at RNAPII genes and nucleosome density at RNAPIII genes [8].

The *S. cerevisiae* chromatin architectural factors and histone modifiers Nhp6A/B are related to the high-mobility group 1 (HMG1) family of small, abundant chromatin proteins that lack sequence specificity of DNA binding, but bend DNA sharply and modulate gene expression [9]. Nhp6 is encoded by two genes, *NHP6A* and *NHP6B*, which are functionally redundant. Consequently, only the *nhp6A nhp6B* double deletion mutant (*nhp6ΔΔ* mutant) is temperature sensitive for growth [10]. Nhp6p is important for activation and repression of transcription of several RNAPII genes [11] and promote transcriptional elongation as part of the FACT complex [12]. Of significance in the context of this paper, Nhp6 is important for expression of the *SNR6* gene, encoding the U6 snRNA transcribed by RNAPIII [13], [14].

The human SWI/SNF subunit BAF57 contains a HMG box domain similar to the one present in Nhp6, which is not found in the yeast complex [15] and the Drosophila BRM component Bap111 is also a HMG-domain protein [16]. In yeast, *NHP6* interacts genetically with both SWI/SNF and RSC [17], both RSC and Nhp6 have a repressive effect on the expression of *CHA1* [3], [11] and data from transcriptome analysis of *swi/snf* and *nhp6ΔΔ* mutants, partly overlap [11]. Furthermore, RSC components interact with Nhp6A *in vitro* and facilitate the loading of Nhp6A onto nucleosomes [17].

A connection between chromatin dynamics and mRNA processing has previously been suggested [18]. The SWI/SNF complex has been linked to alternative pre-mRNA splicing [19], [20]. In higher eukaryotes pre-mRNA splicing is suggested to be a co-transcriptional event [21], [22]. In yeast splicing mainly occurs post-transcriptionally, but initiation of spliceosome assembly and removal of introns from genes with long second exons are probably co-transcriptional events [23], [24]. The spliceosome consists of 5 snRNPs (small nuclear ribonucleoprotein particles (U1, U2, U4, U5, U6)) as well as non-snRNP proteins [25], [26]. Brg1, a subunit of the mammalian orthologue of the yeast SWI/SNF complex interacts with hPrp4, a U5 snRNP-associated kinase [27]. Brm, also a subunit of the mammalian orthologue of the yeast RSC (SWI/SNF) complex, was found upon over-expression to favor inclusion of variant exons in the mRNA and to associate with both U1- and U5-snRNP as well as with coding regions of intron-containing genes [20]. Brm in insect cells was shown to be associated with nascent pre-mRNA's and to regulate the type of alternative transcripts produced [19]. Brm, Brg1 and additional SWI/SNF-related polypeptides associate with chicken supraspliceosomes [28]. Included in the supraspliceosome is the NineTeen Complex (NTC), which functions in spliceosome activation by specifying the interaction of U5 and U6 with pre-mRNA for their stable association with the spliceosome after U1 and U4 dissociation [29], [30].

Here we take a genetic approach and discover an interplay between HMG proteins, chromatin remodeling factors and mRNA maturation. We show that *rsc nhp6ΔΔ* triple mutants accumulate pre-mRNA and demonstrate that *rsc8-ts16 nhp6ΔΔ* cells display low levels of the U4/U6 snRNA dimer and of total U6 snRNA. Thus, a link between chromatin remodelers, architectural factors and mRNA maturation is established.

## Results

### Chromatin remodeling complexes and Nhp6 interact genetically

**Figure 1:**
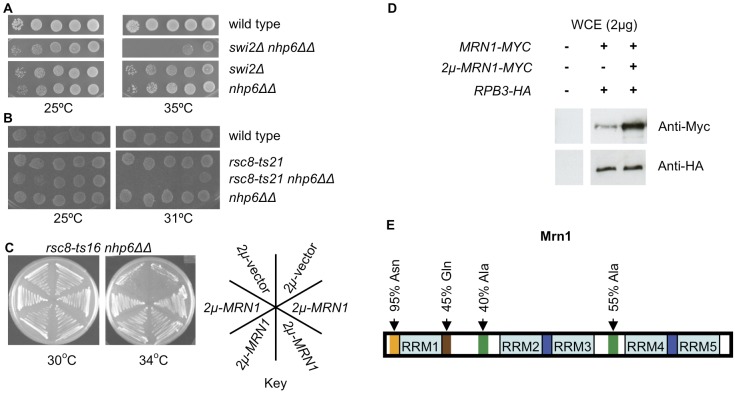
Synthetic sickness of *swi/snf nhp6ΔΔ* and *rsc nhp6ΔΔ* triple mutants is suppressed by *2 µm-MRN1*. (A) and (B) Cells ten-fold serially diluted, spotted on SC plates and incubated for four days. Wild type: SG632; *swi2Δ*: SG418; *nhp6ΔΔ*: SG727; *swi2Δ nhp6ΔΔ*: SG759; wild type: SG358; *rsc8-ts21*: SG359; *nhp6ΔΔ*: SG394; *rsc8-ts21 nhp6ΔΔ*: SG658. Colony rows compared in the same panel derives from one plate. (C) Ability of *2 µm-MRN1* to suppress *rsc8-ts16 nhp6ΔΔ*. Cells streaked on SC-His plates and incubated for four days. Shown on the plates are two transformants containing *2 µm*-vector and four transformants containing *2 µm-MRN1*. *rsc8-ts16 nhp6ΔΔ*: SG657; *2 µm*-vector: pTK839; *2 µm-MRN1*: pTK1395. (D) Western blot analysis to visualize levels of endogenously expressed and *2 µm* expressed Mrn1-Myc. Rpb3-HA serves as a loading control. Untagged strain TG694 and tagged strain SG640 containing either pTK839 or pTK1423. Two µg of whole cell extract was separated on a SDS-PAGE and transferred to a mixed cellulose ester membrane and immunoblotted with anti-HA or anti-Myc antibody as indicated. (E) A schematic representation of the predicted domains and identified regions in Mrn1 (See text for details).

In *Saccharomyces cerevisiae*, the remodeling complex RSC and the architectural factors Nhp6 have a repressive effect on the chromatin structure at the *CHA1* locus [3], [11]. Release of both RSC- and Nhp6-dependent repression results in increased transcript levels of *CHA1* mRNA, suggesting that RSC and Nhp6 co-operate in *CHA1* repression [3], [11]. To identify further relationships between RSC and Nhp6, we tested whether *NHP6* genetically interacts with RSC or SWI/SNF and found that the *swi2Δ nhp6ΔΔ* and *rsc8-ts21 nhp6ΔΔ* triple mutants exhibited a synthetic sickness phenotype compared to their cognate single and double mutants (Figure 1A and Figure 1B). The combination of *rsc* mutations *rsc8-ts16*, *sfh1-1*, *sth1-3ts*, *rsc1Δ* or *rsc2Δ* and *swi/snf* mutations *swi3Δ*, *snf5Δ* or *snf6Δ* with *nhp6ΔΔ* also resulted in reduced growth (Table 1). Thus, the architectural factor Nhp6 shares functionality with RSC and SWI/SNF.

**Table 1:** *2 µm-MRN1* Suppresses *rsc nhp6* and *swi/snf nhp6* Synthetic Sickness.

Complex	Genotype	Synthetic sick	*2 µm-MRN1* suppression	Restrictive temperature
RSC				
	*rsc1Δ nhp6ΔΔ*	Yes	Yes	36°
	*rsc2Δ nhp6ΔΔ*	Yes	No	36°
	*rsc8-ts16 nhp6ΔΔ*	Yes	Yes	34°
	*rsc8-ts21 nhp6ΔΔ*	Yes	ND	31°
	*sfh1-1 nhp6ΔΔ*	Yes	ND	32°
	*sth1-3ts nhp6ΔΔ*	Yes	Yes	35°
SWI/SNF				
	*swi2Δ nhp6ΔΔ*	Yes	Yes	35°
	*swi3Δ nhp6ΔΔ*	Yes	ND	35°
	*snf5Δ nhp6ΔΔ*	Yes	ND	36°
	*snf6Δ nhp6ΔΔ*	Yes	ND	31°

Strains used in Table 1: SG759 (*snf5Δ nhp6ΔΔ*), SG469 (*swi2Δ nhp6ΔΔ*), SG476 (*rsc2Δ nhp6ΔΔ*), SG518 (*rsc1Δ nhp6ΔΔ*), SG657 (*rsc8-ts16 nhp6ΔΔ*), SG658 (*rsc8-ts21 nhp6ΔΔ*), SG659 (*sfh1-1 nhp6ΔΔ*), SG661 (*sth1-3ts nhp6ΔΔ*), SG662 (*snf6Δ nhp6ΔΔ*), SG742 (*swi3Δ nhp6ΔΔ*) and SG759 (*swi2Δ nhp6ΔΔ*). ND = Not determined.

### Multi-copy growth suppression screen of *rsc8-ts16 nhp6ΔΔ* yields *MRN1*

Next we performed a suppression screen of the *rsc8-ts16 nhp6ΔΔ* synthetic sickness phenotype. Using a Yep24-based (*2 µm*) genomic library [31] we isolated *YPL184c* as a multi-copy suppressor (Figure 1C) and named it *MRN1* for multi-copy suppressor of *rsc nhp6*. Western blot analysis of Myc-tagged *2 µm-MRN1* confirmed increased levels of the Mrn1 protein (Figure 1D). Multi-copy *MRN1* was able to suppress the growth defect of all tested *rsc nhp6* and *swi/snf nhp6* triple mutants except *rsc2Δ nhp6ΔΔ* (Table 1). The latter result is likely indicative of the inability of the *rsc2Δ* mutant to maintain *2 µm* plasmids [32].

The Mrn1 protein is predicted to be 612 amino acids long and to contain as many as five RNA Recognition Motifs (RRMs, Figure 1E). Four of these are arranged in pairs and within each pair the RRMs are separated by a short linker (∼23 amino acids, Figure 1E). Present in all kingdoms of life, and most abundantly in eukaryotes, RRM domains are able to bind RNA and also DNA and protein(s) [33]. In addition to the predicted RRM domains, Mrn1 contains an N-terminal region rich in asparagine (91% between amino acids 6 and 28, Figure 1E), a region rich in glutamine (44% between amino acids 98 and 125, Figure 1E) and two regions rich in alanine (40–55% between amino acids 171–189 and amino acids 407–422 respectively, Figure 1E).

### Cellular location of Mrn1

**Figure 2:**
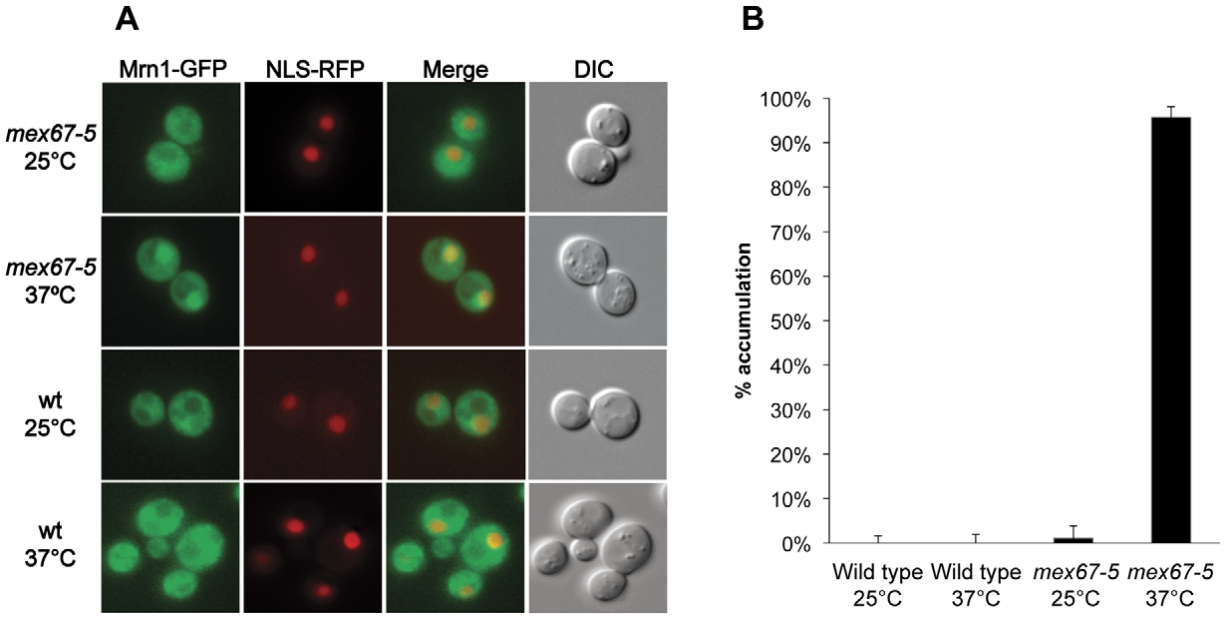
Mrn1-GFP accumulates in the nucleus in a *mex67-5* mutant at 37°C. *MRN1-GFP* and *mex67-5 MRN1-GFP* cells were harvested after growth in SC medium at 25°C or after 30 min incubation at 37°C. For each genotype and growth condition, 100–200 cells were inspected. *Error bars* indicate 95% confidence intervals. SG1008: *mex67-5 MRN1-GFP ADH1p-NLS-yEmRFP::URA3* and SG1010: *MRN1-GFP ADH1p-NLS-yEmRFP::URA3*.

The cellular location of Mrn1-GFP expressed from its genomic location has been reported to be cytoplasmic [34] (http://yeastgfp.ucsf.edu/)). Using the same Mrn1-GFP tagged strain we also observed Mrn1 located primarily in the cytoplasm both at 25°C and 37°C (Figure 2A). However, in these cells we estimated that approximately 5% of Mrn1 is nuclear (M. Lisby, personal communication). To unambiguously detect Mrn1 in the nucleus, we analyzed Mrn1 localization in the temperature-sensitive *mex67-5* mRNA export deficient mutant [35]. In the *mex67-5* genetic background we detected Mrn1-GFP accumulation in the nucleus at 37°C in approximately 95% of the cells (Figure 2A and Figure 2B). This demonstrates that Mrn1 is located both in the nucleus and the cytoplasm.

### Genetic link between chromatin, *MRN1* and mRNA processing

**Figure 3:**
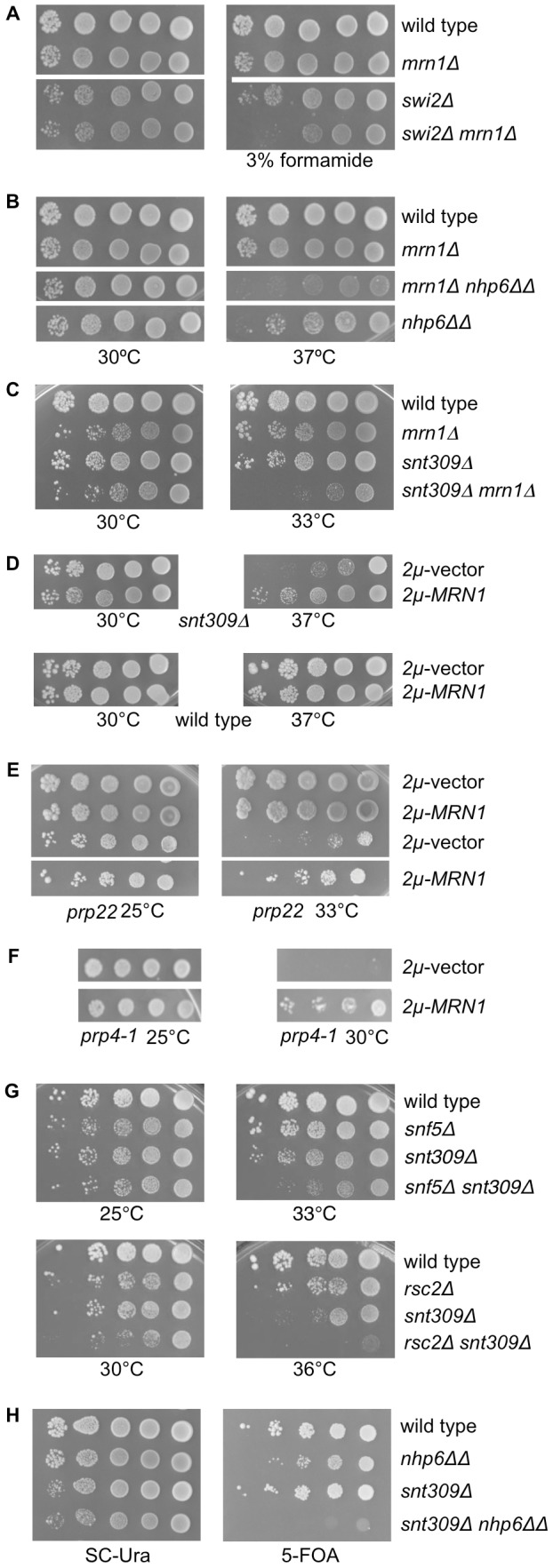
Genetic interactions linking *MRN1* and chromatin mutants to pre-mRNA splicing. (A) *mrn1Δ* is synthetic sick with *swi2Δ*. Cells ten-fold serially diluted, spotted on SC plates or SC plates containing 3% formamide and incubated for four days at 30°. *swi2Δ*: SG418; *mrn1Δ*: SG520; wild type: SG632 and *mrn1Δ swi2Δ*: SG766. (B) *mrn1Δ* is synthetic sick with *nhp6ΔΔ*. Cells ten-fold serially diluted, spotted on SC plates and incubated for four days at the indicated temperatures. *mrn1Δ*: SG520; wild type: SG632; *nhp6ΔΔ*: SG727 and *mrn1Δ nhp6ΔΔ*: SG762. (C) *mrn1Δ snt309Δ* cells are synthetic sick. Cells ten-fold serially diluted, spotted on SC plates and grown at the indicated temperatures for four days. *mrn1Δ*: SG912; wild type: SG632; *snt309Δ*: SG648; *mrn1Δ snt309Δ*: SG920. (D) The temperature sensitivity of *snt309Δ* is suppressed by *2 µm-MRN1*. Cells ten-fold serially diluted, spotted on SC-His plates and incubated for four days at the indicated temperatures. Wild type: SG632; *snt309Δ*: SG648; *2 µm*-vector: pTK839; *2 µm-MRN1*: pTK1395. (E) The temperature sensitivity of *prp22* is suppressed by *2 µm-MRN1*. Cells ten-fold serially diluted, spotted on SC-His plates and incubated for four days at the indicated temperatures. Wild type: SG682; *prp22*: SG840; *2 µm*-vector: pTK839; *2 µm-MRN1*: pTK1423. (F) The temperature sensitivity of *prp4-1* is suppressed by *2 µm-MRN1*. Cells ten-fold serially diluted, spotted on SC-Ura plates and incubated for four days at the indicated temperatures. *prp4-1*: SG845; *2 µm*-vector: pTK51; *2 µm-MRN1*: pTK1386. (G) *snf5Δ* and *rsc2Δ* genetically interacts with *snt309Δ*. Cells ten-fold serially diluted, spotted on SC plates and incubated for four days at the indicated temperatures. *rsc2Δ*: SG417; *snf5Δ*: SG420; wild type: SG632; snt309Δ: SG729; rsc2Δ *snt309Δ*: SG773 and *snf5Δ snt309Δ*: SG774. (H) *snt309Δ* is synthetic lethal with *nhp6ΔΔ*. Cells ten-fold serially diluted, spotted on SC-Ura plates or 5-FOA plates and incubated for four days at 30°C. Wild type: SG865; *nhp6ΔΔ*: SG867; *snt309Δ*: SG868; *snt309Δ nhp6ΔΔ*: SG869; *2 µm*-*NHP6B-URA3*: pTK1382. Colony rows compared in the same panel derives from one plate.

To substantiate the genetic link between *MRN1* and chromatin remodeling complexes, we combined *mrn1Δ* with *swi2Δ* or *nhp6ΔΔ*, respectively. We found that the *mrn1Δ swi2Δ* combination resulted in synthetic sickness on plates containing 3% formamide (Figure 3A), which is known to cause transcriptional stress, and that the *mrn1Δ nhp6ΔΔ* triple deletion was sick at 37°C (Figure 3B). In contrast, the *mrn1Δ snf5Δ* or *mrn1Δ rsc2Δ* combinations did not reveal enhanced growth defects (data not shown).

The presence of RRM domains in Mrn1 could suggest a possible role of the protein in mRNP maturation. Interestingly, an ongoing Synthetic Genetic Array (SGA) screen with *mrn1Δ* as query linked *MRN1* genetically to several splicing deficient mutants (SGA screen to be published elsewhere). Thus, combining *mrn1Δ* and the *snt309Δ* mutant deleted of the NineTeen Complex (NTC) subunit Snt309 resulted in synthetic sickness (Figure 3C). Snt309 associates with the spliceosome simultaneously with or immediately after dissociation of U4 [36] and the *snt309Δ* mutant has a splicing defect that results in the accumulation of intron-containing pre-mRNA at the non-permissive temperature *in vivo* [36]. Also, we found that *2 µm-MRN1* interacted genetically with *snt309Δ* as *2 µm-MRN1* suppressed the ts-phenotype of the *snt309Δ* mutant strain (Figure 3D). The synthetic sickness of *mrn1Δ snt309Δ* indicated that multi-copy *MRN1* suppression of the ts-phenotype of *snt309Δ* mutant reflects relevance for endogenous *MRN1* function. Constanzo et al. recently reported that *mrn1Δ* interacts genetically with *prp4-1*, *prp22*, and *snt309Δ* [37]. Interestingly, *2 µm-MRN1* also suppressed the ts-phenotype of *prp22* (Figure 3E), *prp4-1* (Figure 3F) and *prp3-1* (data not shown). As *2 µm-MRN1* suppressed the *swi/snf* and the *rsc nhp6ΔΔ* triple mutants as well as *snt309Δ* mutant, we examined whether *rsc, swi/snf* or *nhp6ΔΔ* interacted genetically with *snt309Δ* and found that both *snf5Δ snt309Δ* and *rsc2Δ snt309Δ* double mutants were synthetic sick (Figure 3G). In agreement with this, Cairns and co-workers reported genetic interaction between *snt309Δ* and *rsc7Δ* [38]. We also revealed a synthetic lethal interaction between *snt309Δ* and *nhp6ΔΔ* by tetrad analysis. Out of 21 tetrads 12 did not contain the triple mutant and the remaining 9 each had 3 viable spores and the missing spore would have been the triple mutant (data not shown). To establish that *snt309Δ* and *nhp6ΔΔ* indeed are synthetic lethal, an *snt309Δ nhp6ΔΔ* heterozygous diploid was transformed with a *URA3* containing plasmid expressing *NHP6B*. After dissection, genotype verification, and spot assay we found that *snt309Δ nhp6ΔΔ* spores were unable to grow on 5-FOA (Figure 3H). However, Snt309p and Mrn1p are not functionally redundant as high-copy *SNT309* did not suppress the growth defect of the *rsc8-ts16 nhp6ΔΔ* triple mutant (data not shown). In addition, Mrn1 does not share genetic functionality with the RRM-containing Prp24 which mediates the re-annealing of the U4/U6 dimer [39] as high-copy *MRN1* did not complement a *prp24Δ* mutant: dissection of 15 tetrads of a *prp24Δ* heterozygous diploid harboring *2 µm-MRN1* (pTK1395) all segregated 2∶0 for viability. In conclusion, the genetic interactions shown in Figure 3 suggest a role for Mrn1, RSC, SWI/SNF and architectural factors in mRNA maturation.

### *rsc8-ts16 nhp6ΔΔ* cells accumulate intron-containing pre-mRNA

**Figure 4:**
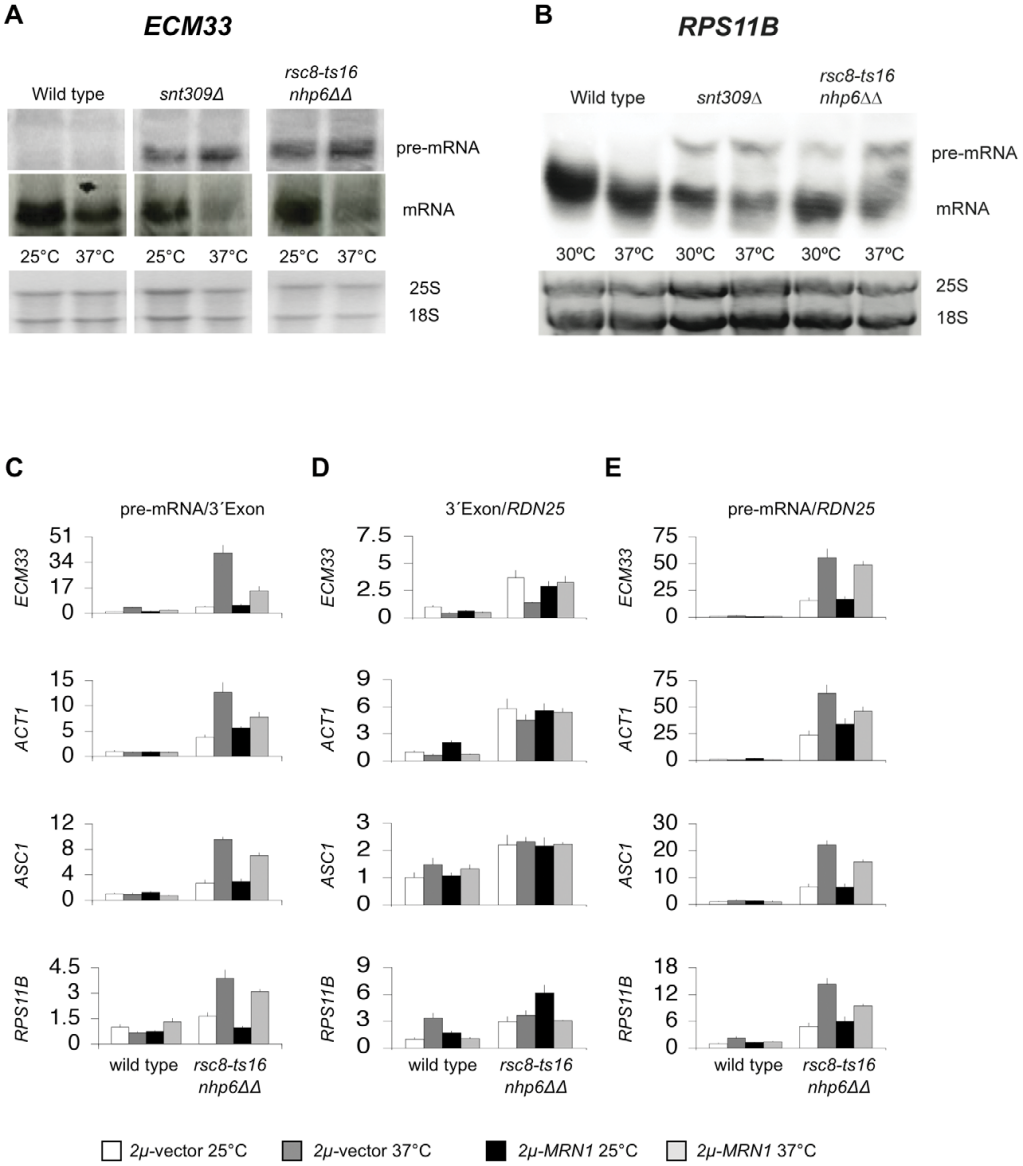
*rsc8-ts16 nhp6ΔΔ* cells accumulate unspliced transcripts. (A) Northern blot analysis was done with total RNA isolated from logarithmically SC-His growing cells at 25°C or after a two hour shift at 37°C. Total RNA was electrophoresed in a 0.25 M formaldehyde agarose gel, blotted and hybridized with specific ^32^P-labeled probes. The probe was either intron-specific or 3′ exon-specific, respectively, for the *ECM33* RNA (see Figure S1). Ethidium bromide staining of the 18S and 25S rRNA is shown as a loading control. (B) Northern blot analysis was done with total RNA isolated from logarithmically SC-His growing cells at 30°C or after a two hour shift at 37°C. The probe was specific for both the *RPS11B* pre-mRNAand for the *RPS11B* mRNA (see Figure S1). Ethidium bromide staining of the 18S and 25S rRNA is shown as a loading control. (C), (D) and (E) Total RNA isolated from logarithmically SC-His growing cells at 25°C or after a two-hour shift at 37°C amplified by RT-qPCR with *ECM33-*, *ACT1-*, *ASC1-*, *RPS11B-* or *RDN25-*specific primers. (C) The ratio intron-3′exon junction RT-PCR-amplificate/3′exon RT-PCR-amplificate. (D) The ratio 3′exon RT-PCR-amplificate/*RDN25* RT-PCR-amplificate. (E) The ratio intron-3′exon junction RT-PCR-amplificate/*RDN25* PCR-amplificate. The ratio in wild type cells at 25°C was arbitrarily set to 1. Wild type: SG632; *rsc8-ts16 nhp6ΔΔ*: SG657; *2 µ*-vector: pTK839; *2 µ-MRN1*: pTK1423.

To analyze if the *rsc nhp6* mutation influences the amounts of intron-containing pre-mRNA *in vivo* we isolated total RNA from *rsc8-ts16 nhp6ΔΔ* and *snt309Δ* (as control) mutant strains grown at 25°C and after a two-hour incubation at 37°C. A Northern blot was probed for the intron-containing *ECM33* pre-mRNA and the *ECM33* 3′ exon mRNA (Figure 4A – see Figure S1 for position of probes). Increased levels of pre-mRNA were observed in both mutants at 25°C and this increase was exacerbated after a two hour incubation at 37°C. We also observed that the amount of mature *ECM33* mRNA at 37°C was strongly decreased in the two mutant strains as compared to wild type. The same analysis of the *RPS11B* pre-mRNA and mRNA also revealed that pre-mRNA levels in both mutants at 30°C were increased compared to the wild type and the levels of both pre-mRNAs were further increased after two hours incubation at 37°C. Again, the increase in *RPS11B* pre-mRNA were accompanied by a decrease in mature mRNA (Figure 4B). To extend the analysis we measured the ratio of pre-mRNA to 3′ exon mRNA by Reverse Transcriptase quantitative-PCR (RT-qPCR) of *ECM33* transcripts. Indeed, the *rsc8-ts16 nhp6ΔΔ* triple mutant had increased *in vivo* pre-mRNA/3′exon ratio of the *ECM33* transcript already at 25°C and this effect was dramatically enhanced after two hours at 37°C (Figure 4C). Although a small (1.5-fold) increase in overall transcription of *ECM33* in the *rec8-ts16 nhp6ΔΔ* mutant was seen, the 55-fold relative increase in the unspliced *ECM33* pre-mRNA at 37°C is 36 times higher than that of the relative increase in total *ECM33* mRNA (compare Figure 4D and Figure 4E). Interestingly, the pre-mRNA accumulation phenotype at 37°C of *rsc8-ts16 nhp6ΔΔ* cells was partly suppressed by *2 µm-MRN1* (Figure 4C). Similarly, RT-qPCR analysis of the three intron-containing genes *ACT1*, *ASC1* and *RPS11B* in the *rsc8-ts16 nhp6ΔΔ* mutant revealed an accumulation of their pre-mRNA's at 25°C and exceedingly more so after incubation at 37°C for 2 hours (Figure 4C). Again, the relative increase in total RNA levels at 37°C was lower (2–5-fold) than the relative increase in pre-mRNA levels (14–60-fold) (compare Figure 4D and Figure 4E). Furthermore, overexpressed Mrn1 modestly suppressed the accumulation of *ACT1*, *ASC1*, and *RPS11B* pre-mRNA at 37°C (Figure 4C). Analyses of all four intron-containing transcripts in the *rsc1Δ nhp6ΔΔ* and *snt309Δ* mutants revealed a similar accumulation of pre-mRNA at 25°C and exceedingly more so after incubation at 37°C for two hours (Figure S2). Importantly, accumulation of *ECM33*, *ACT1*, *ASC1*, *RPS11B* pre-mRNAs did not generally occur in single *rsc* mutants or in the double *nhp6* deletion strain (Figure S3). Thus, *rsc nhp6* triple mutants exhibited an mRNA maturation deficiency, which was aggravated after a two hour incubation at 37°C. In addition to suppression of the temperature sensitivity of the *rsc nhp6* triple and *snt309Δ* strains, Mrn1 over-expression also modestly suppressed the pre-mRNA accumulation exhibited by the mutants.

### Reduced U4/U6 dimer snRNA levels in *rsc nhp6ΔΔ* cells

**Figure 5:**
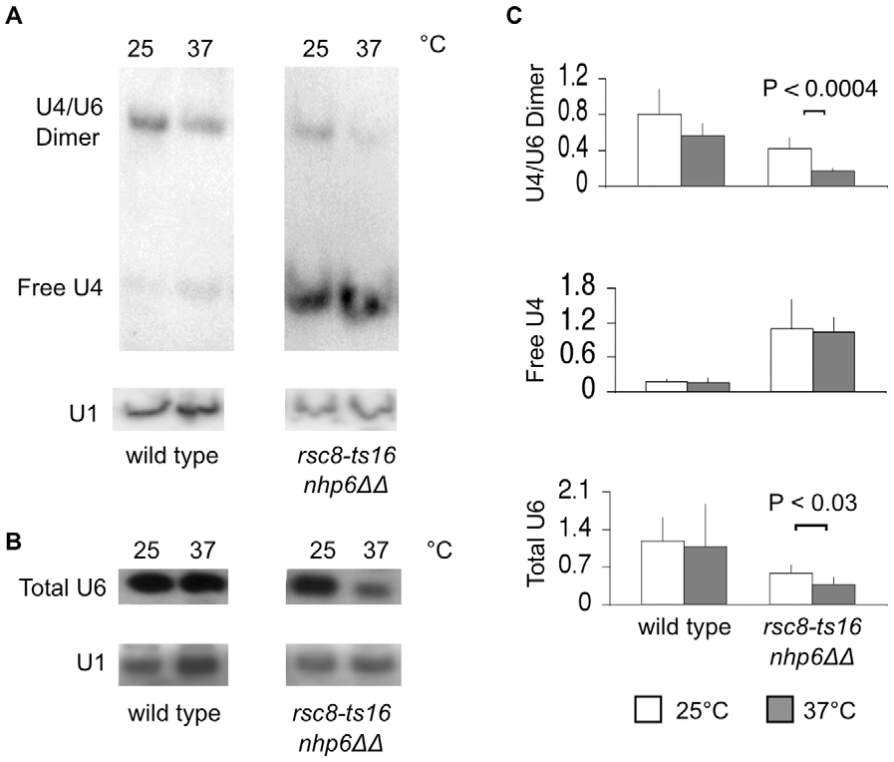
U4/U6 dimer, free U4 and total U6 snRNA levels in *rsc8-ts16 nhp6ΔΔ* cells. Total RNA prepared from logarithmically SC-His growing cells at 25°C or after a two-hour shift at 37°C. (A) RNA was fractionated on a non-denaturing 6% polyacrylamide gel, blotted and hybridized with a U4 specific probe. After analysis the membrane was stripped and re-probed with a U1 specific probe. (B) RNA was fractionated on a denaturing 6% polyacrylamide gel, blotted and hybridized with a U6 specific probe. After analysis the membrane was stripped and re-probed with a U1 specific probe. (C) Quantification of U4/U6 dimer, free U4 and total U6 snRNA amounts relative to U1 snRNA based on quantification of Storm Images from at least five individual experiments. Wild type: SG632; *rsc8-ts16 nhp6ΔΔ*: SG657.

In wild type cells U6 snRNP is in excess of U4 snRNP, but reduced levels of U6 is a common phenotype in strains with mutations in genes encoding U6, U4/U6, or tri-snRNP components including Prp3, Prp4, Prp19, Prp24, Prp38 and Lsm proteins [40], [41], [42], [43], [44], [45], [46]. Apparently, in these mutant strains the U4/U6 complex is destabilized. Specifically, in *snt309Δ* mutant cells the U4/U6 dimer is destabilized, resulting in accumulation of free U4 and decreased levels of total U6 and in failure of spliceosome recycling due to impaired U4/U6 biogenesis [47]. This is underscored as over-expressed U6 suppresses the ts-phenotype of *snt309Δ* [47]. In addition, we found that *2 µm-SNR6* restored growth of the *rsc8-ts16 nhp6ΔΔ* triple mutant (data not shown). To determine the levels of the U4/U6 dimer, free U4 and total U6 in *rsc8-ts16 nhp6ΔΔ* cells total RNA was fractionated both on non-denaturing and denaturing polyacrylamide gels for Northern analysis with U4 and U6 specific probes, respectively. The *rsc8-ts16 nhp6ΔΔ* cells had decreased amounts of the U4/U6 dimer and accumulated free U4 at 25°C (Figure 5A). Interestingly, the amount of U4/U6 dimer was further decreased after a two-hour incubation at 37°C. In the mutant total U6 snRNA levels were also reduced after two hours at 37°C (Figure 5B). To quantify the snRNA levels we performed five independent experiments and normalized the U4 and U6 data to the same blots re-probed for U1 snRNA. First, the *rsc8-ts16 nhp6ΔΔ* strain showed a more than 5-fold increase in free U4; second, at 25°C the mutant strain had a 2-fold decrease in the levels of U4/U6 and total U6; third, a two-hour shift to 37°C resulted in a further, significant 1–2-fold reduction in U4/U6 and total U6 levels (Figure 5C). We conclude that *rsc8-ts16 nhp6ΔΔ* cells contain low levels of U4/U6 dimer snRNA, of total U6 snRNA and accumulate free U4 snRNA at 25°C and that the low levels of U4/U6 dimer and of total U6 is further aggravated after a two-hour shift at 37°C.

### U4/U6 dimer and total U6 snRNA levels are unchanged after a two-hour transcriptional shutdown

**Figure 6:**
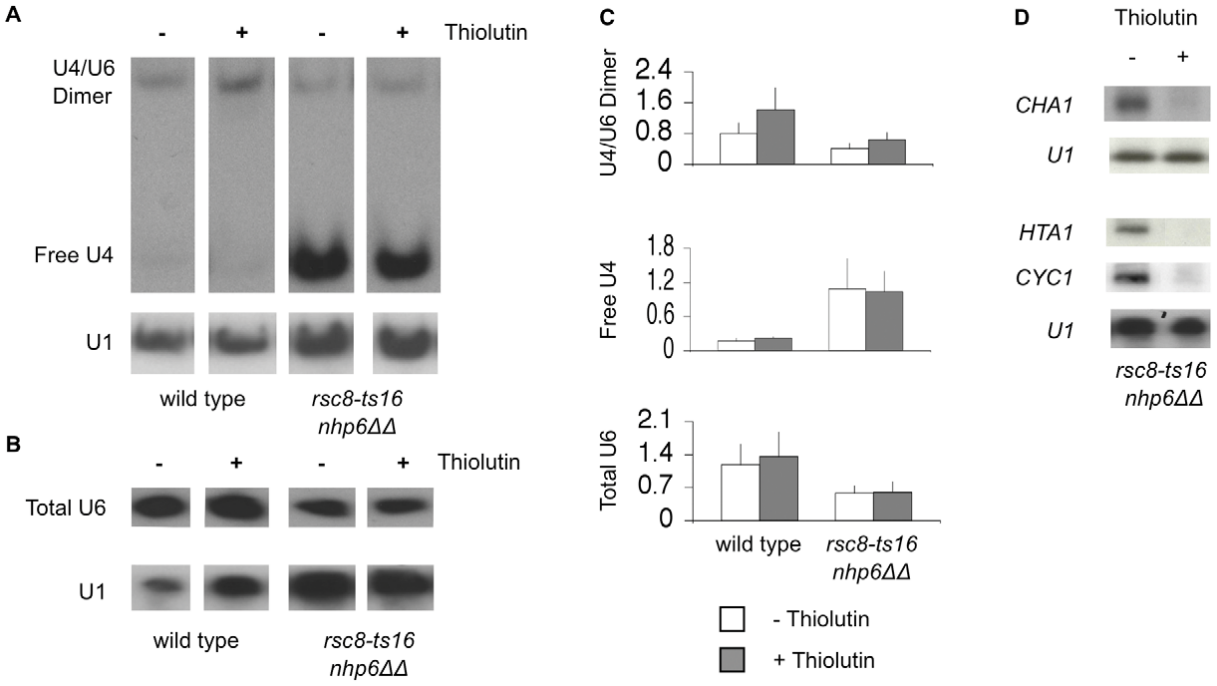
U4/U6 dimer, free U4 and total U6 snRNA levels remain stable after two hours of transcriptional shutdown. Total RNA prepared from logarithmically SC-His growing cells at 25°C or after two hours growth in the presence of 5 µg/ml Thiolutin at 25°C. (A) RNA was fractionated on a non-denaturing 6% polyacrylamide gel, blotted and hybridized with a U4 specific probe. After analysis the membrane was stripped and re-probed with a U1 specific probe. (B) RNA was fractionated on a denaturing 6% polyacrylamide gel, blotted and hybridized with a U6 specific probe. After analysis the membrane was stripped and re-probed with a U1 specific probe. (C) Quantification of U4/U6 dimer, free U4 and Total U6 snRNA amounts relative to U1 snRNA based on quantification of Storm Images from at least four individual experiments. (D) Total RNA was isolated from logarithmically SC-His growing *rsc8-ts16 nhp6ΔΔ* cells at 25°C and electrophoresed in a 0.25 M formaldehyde agarose gel or in a denaturing 6% polyacrylamide gel, blotted and hybridized with gene-specific ^32^P-labeled probes. *rsc8-ts16 nhp6ΔΔ* : SG657.

Both RSC and Nhp6 are involved in transcriptional regulation of RNAPIII transcribed genes and high-copy *SNR6* suppresses the growth defect of *nhp6ΔΔ* double mutants [14], [48]. Therefore, it was important to determine if the observed reduction in U4/U6 dimer and total U6 snRNA levels in the triple mutant was an effect of the *rsc nhp6* mutations to reduce *SNR6* transcription. In this case the drop in U4/U6 and total U6 snRNA content would just reflect *SNR6* RNA turnover. We addressed this question by determining U4/U6 dimer, free U4 and total U6 stability in *rsc8-ts16 nhp6ΔΔ* cells after growth for two hours in the presence of thiolutin. The antifungal agent thiolutin efficiently inhibits all three yeast polymerases both *in vivo* and *in vitro* [49], [50]. Total RNA was isolated from cells treated with thiolutin for two hours and the levels of U4/U6, free U4, and total U6 was determined by Northern blotting. The amount of U4/U6 dimer, free U4 and total U6 was unchanged after two hours of incubation with thiolutin both in the wild type and more importantly, also in the *rsc8-ts16 nhp6ΔΔ* strain (Figure 6A and Figure 6B). Quantification of four independent experiments confirmed this result (Figure 6C). In contrast, as expected the levels of three tested mRNA's were drastically reduced under the same growth conditions (Figure 6D). Thus, efficiently shutting down RNA polymerase III transcription by the polymerase inhibitor thiolutin for two hours did not influence the levels of U4/U6 dimer or total U6 snRNA neither in the wild type nor in the *rsc8-ts16 nhp6ΔΔ* strain indicating that the decrease in snRNA levels is not due to impaired transcription of the *SNR6* gene, but only observed in cells with specific splicing-deficient conditions.

## Discussion

In this study we have utilized a genetic approach to study the functional interplay between the chromatin remodeling complexes RSC or SWI/SNF and the architectural factors Nhp6. We found that *rsc-* or *swi*/*snf* mutations in combination with *nhp6* double deletion results in synthetic sickness. Interestingly, we found that *rsc nhp6* triple mutants accumulate pre-mRNA, strongly suggesting a defect in pre-mRNA maturation. The defect in pre-mRNA maturation is underscored as *rsc8-ts16 nhp6ΔΔ* cells contained low levels of U4/U6 dimer and total U6 snRNA as well as high amounts of free U4 snRNA. Further, incubation at 37°C for two hours dramatically enhanced the accumulation of pre-mRNA in *rsc nhp6ΔΔ* cells. This is substantiated as *rsc8-ts16 nhp6ΔΔ* cells contained significantly reduced amounts of U4/U6 dimer and total U6 after two hours incubation at 37°C. The reduction in U4/U6 dimer and total U6 was not due to deficient *SNR6* transcription as a two-hour shutdown of SNR6 transcription induced by thiolutin did not result in reduced amounts of U4/U6 dimer or total U6 indicating that these RNAs are very stable. In agreement with this result, U6 snRNA have previously been reported to be very stable unless in a splicing deficient mutant background [40]. For example, temperature inactivation of the known U6 (or U4/U6) snRNP associated factors Prp3, Prp4, Prp6, Prp24 or the NTC or Prp38 splicing factors lead to a decrease in U6 snRNA levels [40], [43], [44], [45], [46], [47]. Apparently, in these mutant strains the U4/U6 complex is destabilized, perhaps exposing the U6 snRNA to intracellular nuclease attack. Furthermore, Moenne *et al.* [51] observed only a slight decrease in total U6 snRNA level after a five hour inactivation of a temperature-sensitive RNAPIII mutant. Accordingly, a two-hour shift to 37°C reduces U4/U6 dimer and total U6 snRNA levels in *rsc8-ts16 nhp6ΔΔ* cells as a consequence of their mRNA processing defect and not as a consequence of deficient transcription of *SNR6*. We did not see a general accumulation of unspliced mRNA for the tested transcripts after a two hour incubation at 37°C in the *rsc1Δ* or *rsc8-ts16* single mutants, or in the *nhp6ΔΔ* double mutant. However, the *rsc* and *swi/snf* single mutants, and the *nhp6ΔΔ* double mutant might harbor potential splicing defects. In support of this notion we observed that combining *rsc2Δ* or *snf5Δ* with the NTC splicing mutant *snt309Δ* resulted in synthetic sickness and that a *snt309Δ nhp6ΔΔ* triple mutant is synthetic lethal. In conclusion, the combination of mutations in RSC and chromatin architectural factors results in a severe defect in pre-mRNA maturation.

We identified the RNA-binding protein Mrn1 as a multi-copy suppressor of the synthetic sickness of the *rsc8-ts16 nhp6ΔΔ* mutant. Mrn1 is predicted to contain five RRM domains present in many RNA-binding proteins taking part in all mRNA co- and post-transcriptional processing events [33]. Recently, Hogan et al. [52] reported that Mrn1 is an RNA-binding protein and interacts with 378 RNAs including *ECM33* and *ACT1*. Our genetic interaction analysis of *MRN1* and *2 µm-MRN1* lead to the discovery that *rsc nhp6ΔΔ* cells display a splicing deficient phenotype as discussed above. In addition, the genetic analysis might also suggest a role of Mrn1 in pre-mRNA maturation. Over-expression of Mrn1 suppressed the ts-phenotype of the *rsc nhp6* triple mutants as well as that of the NTC subunit mutant *snt309Δ*. Combining *mrn1Δ* with *snt309Δ* resulted in synthetic sickness, but Mrn1 and Snt309 are not functionally redundant as only *2 µm-MRN1*, and not *2 µm-SNT309* suppresses the *rsc8-ts16 nhp6ΔΔ* triple mutant phenotype. Additionally, Mrn1 does not share genetic functionality with the RRM-containing Prp24. Prp24 mediates the re-annealing of the U4/U6 dimer [39], but *2 µm-PRP24* does not suppress the ts-phenotype of *snt309Δ* cells [47] and *2 µm-MRN1* does not suppress lethality of a *prp24Δ* mutant. However, the suppression is specific, at least to a certain degree, as over-expression of either Pub1 or human PTB, two RNA binding proteins with two pairs of RRM domains arranged as those of Mrn1, does not suppress the growth defect of the *rsc8-ts16 nhp6ΔΔ* triple mutant (J. Christiansen and S. Holmberg, unpublished data). Work in progress in our lab is trying to identify a specific event in the mRNA processing pathway where Mrn1 functions.

The observed pre-mRNA accumulation in the *rsc nhp6* triple mutants can be explained in several ways. The lack of RSC/Nhp6 activity concomitantly might influence transcription of splicing factor-encoding genes leading to the observed pre-mRNA accumulation and U4/U6 destabilization. It is also possible that the primary splicing block imposed by the *rsc8-ts16 nhp6ΔΔ* mutant results from the failure of splicing complexes to assembly or function properly. Thus, RSC and Nhp6 might be required for generating the correct chromatin state required for proper spliceosome assembly thereby affecting mRNA processing. Recent studies document connections between chromatin and splicing. The mammalian orthologue of the RSC complex, hSWI/SNF subunit Brm, was found to associate with several components of the spliceosome as a regulator of alternative splicing in several mammalian cell types [20]. Likewise, Brm and several hSWI/SNF subunits were shown to associate with chicken supraspliceosomes [28]. In yeast only very few genes contain more than one intron, and although it has been reported that most splicing is post-transcriptionally, recruitment of U1 is a co-transcriptional event at probably all genes [24]. One possibility is that *rsc nhp6* and *swi/snf nhp6* cells are deficient in the process of co-transcriptional recruitment of the pre-spliceosome. Batsché *et al.* [20] showed that Brm interacts *in vivo* with both U1 and U5 snRNPs and suggested that hSWI/SNF is involved in recruitment of the splicing machinery. Tyagi *et al.* [19] recently showed that Brm interacts directly with nascent pre-mRNP's and suggest that Brm post-transcriptionally regulates the type of alternative transcript produced. Whether RSC, SWI/SNF and/or Nhp6 factors can be loaded onto pre-mRNA in yeast remains to be elucidated.

## Materials and Methods

### Media, strains and genetic methods

Yeast extract-peptone-dextrose (YPD) medium, synthetic minimal (SD) medium, synthetic complete (SC) and SC lacking specific amino acids were prepared as described previously [53]. Standard yeast methods were used for dissection, sporulation, mating and replica plating. Lithium acetate transformation was employed [54]. Yeast strains are listed in Table 2, plasmids in Table 3, and oligonucleotides in Table 4.

**Table 2:** Yeast Strains Used in This Study.

Strain	Genotype	Source or reference
SG304	RJY6009: *MATα ura3 leu2 his3 trp1 lys2 Δnhp6A::URA3 Δnhp6B::LEU2*	[58]
SG306	RJY6012: *MATα ura3 leu2 his3 trp1 lys2 Δnhp6A::ura3 Δnhp6B::LEU2*	[58]
SG312	CY332: *MATα snf6Δ ura3-52 leu2-Δ1 his3-Δ200 trp1-Δ1 lys2-801 ade2-101*	Craig Peterson
SG350	BLY49: *MAT* **a** *sth1-3ts his3-Δ200 ura3-52 ade2-101*	[59]
SG358	MCY3839: *MATα his3 leu2 ura3 lys2*	[60]
SG359	MCY3888: *MATα his3 leu2 ura3 lys2 rsc8-ts21*	[60]
SG360	MCY3890: *MAT* **a** *his3 leu2 ura3 ade2 trp1 can1 rsc8-ts16*	[60]
SG394	*MAT* **a** *his3 leu2 ura3 trp1 nhp6A::URA3 nhp6B::LEU2*	José Moreira
SG416	BY4741 *MAT* **a** *leu2Δ0 his3Δ1 ura3Δ0 met15Δ0 rsc1::KANMX*	Euroscarf
SG417	BY4741 *MAT* **a** *leu2Δ0 his3Δ1 ura3Δ0 met15Δ0 rsc2::KANMX*	Euroscarf
SG418	BY4741 *MAT* **a** *leu2Δ0 his3Δ1 ura3Δ0 met15Δ0 swi2::KANMX*	Euroscarf
SG420	BY4741 *MAT* **a** *leu2Δ0 his3Δ1 ura3Δ0 met15Δ0 snf5::KANMX*	Euroscarf
SG462	*MAT* ^1^ *his3 leu2 ura3 snf5::KANMX nhp6a::URA3 nhp6b::LEU2*	This study
SG476	*MAT* ^1^ *his3 leu2 ura3 rsc2::KANMX nhp6A::URA3 nhp6B::LEU2*	This study
SG485	DY7103 *MATα ade2 can1 his3 leu2 trp1 ura3 RPB3-HA(3)::KANMX*	David J. Stillman
SG518	*MAT* ^1^ *his3 leu2 ura3 rsc1::KANMX nhp6A::ura3 nhp6B::LEU2*	This study
SG520	BY4742 *MATα his3 leu2 ura3 lys2 mrn1::KANMX*	Euroscarf
SG605	*MAT* **a** *his3-11,15 leu2-3,112 ura3 ade2-1 trp1can1-100 MRN1-13Myc::KANMX*	This study
SG632	BY4741 *MAT* **a** *leu2Δ0 his3Δ1 ura3Δ0 met15Δ0*	Euroscarf
SG633	BY4742 *MATα leu2Δ0 his3Δ1 ura3Δ0 lys2Δ0*	Euroscarf
SG640	*MATα ade2 his3 leu2 trp1 ura3 lys RPB3-HA::KANMX MRN1-MYC::KANMX*	This study
SG648	BY4741 *MAT* **a** *leu2Δ0 his3Δ1 ura3Δ0 met15Δ0 snt309::KANMX*	Euroscarf
SG657	*MATα his3 leu2 ura3 lys2 trp1 ade2 rsc8-ts16 nhp6A::ura3 nhp6B::LEU2*	This study
SG658	*MAT* ^1^ *his3 leu2 ura3 lys2 trp1 rsc8-ts21 nhp6A::URA3 nhp6B::LEU2*	This study
SG659	*MAT* ^1^ *trp1 leu2 his3 ura3 sfh1-1::HIS3 nhp6A::URA3 nhp6B:: LEU2*	This study
SG661	*MAT* ^1^ *his3 leu2 ura3 sth1-3ts nhp6A::URA3 nhp6B::LEU2*	This study
SG662	*MAT* ^1^ *trp1 leu2 his3 ura3 ade2 lys2 snf6Δ nhp6A::URA3 nhp6B::LEU2*	This study
SG682	W303 *MAT* **a** *his3 leu2 ura3 ade2 trp1 can1*	Brad Cairns
SG727	*MAT* **a** *leu2Δ0 his3Δ1 ura3Δ0 met15Δ0 nhp6A::URA3 nhp6B::LEU2*	This study
SG729	*MATα leu2Δ0 his3Δ1 ura3Δ0 lys2Δ0 snt309::KANMX*	This study
SG736	*MATα leu2 his3 ura3 mex67-5 MRN1-GFP::HIS3*	This study
SG737	*MATα leu2 his3 ura3 MRN1-GFP::HIS3*	This study
SG742	*MAT* ^1^ *his ura leu trp lys swi3::KANMX nhp6A::URA3 nhp6B::LEU2*	This study
SG759	*MAT* **a** *leu2Δ0 his3Δ1 ura3Δ0 met15Δ0 swi2::KANMX nhp6A::URA3 nhp6B::LEU2*	This study
SG762	*MATα leu2Δ0 his3Δ1 ura3Δ0 mrn1::KANMX nhp6A::URA3 nhp6B::LEU2*	This study
SG766	*MAT* ^1^ *leu2Δ0 his3Δ1 ura3Δ0 lys2Δ0 mrn1::KANMX swi2::KANMX*	This study
SG773	*MAT* ^1^ *leu2Δ0 his3Δ1 ura3Δ0 lys2Δ0 snt309::KANMX rsc2::KANMX*	This study
SG774	*MAT* ^1^ *leu2Δ0 his3Δ1 ura3Δ0 lys2Δ0 snt309::KANMX snf5::KANMX*	This study
SG840	*MAT* **a** *prp22 ade2-101 his3Δ200 ura3-52 tyr1*	[61]
SG845	*MAT* **a** *prp4-1 leu2 ura3-52*	J. Beggs
SG865	*MAT* ^1^ *leu2Δ0 his3Δ1 ura3Δ0 met15Δ0*+pTK1382	This study
SG867	*MAT* ^1^ *leu2Δ0 his3Δ1 ura3Δ0 met15Δ0 lys2Δ0 nhp6A::KANMX nhp6B::KANMX*+pTK1382	This study
SG868	*MAT* ^1^ *leu2Δ0 his3Δ1 ura3Δ0 met15Δ0 snt309::KANMX*+pTK1382	This study
SG869	*MAT* ^1^ *leu2Δ0 his3Δ1 ura3Δ0 met15Δ0 lys2Δ0 nhp6A::KANMX nhp6B::KANMX snt309::KANMX*+pTK1382	This study
SG912	BY4742 *MATα his3 leu2 ura3 lys2 mrn1:: ClonNAT*	This study
SG920	*MAT* ^1^ *leu2Δ0 his3Δ1 ura3Δ0 lys2Δ0 mrn1::ClonNAT snt309::KANMX*	This study
SG1008	*MATα leu2 his3 ura3 mex67-5 MRN1-GFP::HIS3 ADH1p-NLS-yEmRFP::URA3*	This study
SG1010	*MATα leu2 his3 ura3 MRN1-GFP::HIS3 ADH1p-NLS-yEmRFP::URA3*	This study
TG693	*MATα his3 leu2 ura3 trp1 ade2 can1 sfh1-1::HIS3*	[62]
TG694	BLY46-2: *MATα his3 leu2 ura3 trp1 ade2 can1*	[62]

*MAT* ^1^: The mating type has not been determined.

**Table 3:** Plasmids Used in This Study.

Name	Genotype	Source or reference
pTK51	Yep24: *2 µm-URA3-Amp^r^*	[63]
pTK839	pRS423: *2 µm-HIS3-Amp^r^*	[64]
pTK1259	pFA6a: *13Myc-KANMX6*	[65]
pTK1382	*2 µm-URA3-Amp^r^-NHP6B*	This study
pTK1385	*2 µm-URA3-Amp^r^-MRN1*	This study
pTK1386	*2 µm-URA3-Amp^r^-MRN1*	This study
pTK1395	*2 µm-HIS3-Amp^r^-MRN1*	This study
pTK1423	*2 µm-HIS3-Amp^r^-MRN1-MYC*	This study
pML96	*URA3-Amp^r^-NLS-yEmRFP*	M. Lisby

**Table 4:** Oligonucleotides Used in This Study.

Oligonucleotides for RT-PCR and RT-qPCR	
Name	Sequence
*ACT1* (intron-exon2): Act1c	5′ GGTCCCAATTGCTCGAGAGATTTC 3′
*ACT1* (intron-exon2): Act1d	5′ CGGCTTTACACATACCAGAACCG 3′
*ACT1* (3′exon): Act1e	5′ GCCTTCTACGTTTCCATCCAAGCC 3′
*ACT1* (3′exon): Act1f	5′ GGCGTGAGGTAGAGAGAAACCAGC 3′
*ASC1* (intron-exon2): Asc1a	5′ CTCTGCTCTTCTCTTTACTCG 3′
*ASC1* (intron-exon2): Asc1b	5′ GTTGATGTTGGAGTTGTGACC 3′
*ASC1* (3′exon): Asc1c	5′ CCCAGACGGAACTTTGATTGC 3′
*ASC1* (3′exon): Asc1d	5′ GCAGCAGCCAACCAGTATCTG 3′
*ECM33* (intron-exon2): Ecm33a	5′ TACATGTATAAATCGATCGGG 3′
*ECM33* (intron-exon2): Ecm33b	5′ CCAACAATGGTACTACAACCG 3′
*ECM33* (3′exon): Ecm33c	5′ GGTGGTGGTTTCATCATTGC 3′
*ECM33* (3′exon): Ecm33e	5′ GCACCACCTCTAACAGACTTC 3′
*RPS11B* (intron-exon2): Rps11a	5′ AACCGCCACGACACAGTTAACG 3′
*RPS11B* (intron-exon2): Rps11b	5′ CTTGGAAGTCTTGACCTTTGG 3′
*RPS11B* (3′exon): Rps11c	5′ CCGTGGTAAGATCTTGACCG 3′
*RPS11B* (3′exon): Rps11d	5′ GGAATGTAATGCAAGTAAGC 3′
*RDN25*: Rdn25-1a	5′ CGACGTAAGTCAAGGATGCTGGC 3′
*RDN25*: Rdn25-1b	5′ CATCAGGATCGGTCGATTGTGC 3′

Oligonucleotides used in PCR amplification of a template for Northern blot probes	
Name	Sequence
*ECM33* (intron): Ecm33g	5′ CCTCACAAATCTCGAGTAGATTC 3′
*ECM33* (intron): Ecm33h	5′ TTAGTATTCCCGATCGATTTATACATG 3′
*ECM33* (3′exon): Ecm33c	5′ GGTGGTGGTTTCATCATTGC 3′
*ECM33* (3′exon): Ecm33f	5′ CGACACCCATGAATGAAGTGGCTGG 3′
*RPS11B:* oMiT138	5′ AGCAGCAGAGACCTTGACGA 3′
*RPS11B:* oMiT138	5′ AGCAGCAGAGACCTTGACGA 3′
U1: Snr19a	5′ GCGGAAGGCGTGTTTGCTGACG 3′
U1: Snr19b	5′ GGCAACG AGCAAAGTTGAGACTGC 3′
Cha1op	5′ CCCCGGAAAGGCTTCTGC 3′
Cha1ned	5′ GAGGAGCCGCCACAAGC 3′
Hta1e	5′ GGTGGTAAAGGTGGTAAAGCTGG 3′
Hta1f	5′ TTGAGAAGCCTTGGTAGCCTTGG 3′
Cyc1a	5′ ATGACTGAATTCAAGGCCGGTTC 3′
Cyc1b	5′ GTAGGTAATTAAGTCGTTTCTGTC 3′

Oligonucleotides used directly as template for Northern blot probes	
Name	Sequence
U4: Snr14	5′CCGAGATTGTGTTTTTGCTGGTTGAAAATTTAATTATAAACCAGACCGTCTCCTCATGGTCAATTCGGTGTTCG 3′
U6: Snr6a	5′GTTCGCGAAGTAACCCTTCGTGGACATTTGGTCAATTTGAAACAATACAGAGATGATCAGCAGTTCCCC 3′

Oligonucleotides used in the amplicification of Myc-Tagged DNA fragment	
Name	Sequence
MYCc	5′TGGAAAGCGGGCAGTGAGCGCAACGCAATTAATGTGAGTACCTCACTCCGGATGTGATGTGAGAACTGTATCCTAGC 3′
MYCab	5′CTTTGGCAAGGATAGATGTGGTAACGTCCCCCACCAATCACGTCGGATCCCCGGGTTAATTAACGG 3′
MYCa	5′CGATGATAAAGATGATAATGTTGCTAACGGTGAGAAACTTGCAGATCGCAGTGGACGAATCGACAGCAGTATAGCGACC 3′
Sfh1d	5′ CCATGAGTGACGACTGAATCCGG 3′
Sfh1e	5′CGCGATAATGTCGGGCAATCAGG 3′

### Multi-copy suppressor screen

Strain SG657 (*rsc8-ts16 nhp6ΔΔ*) was transformed with a Yep24 based yeast genomic library [31]. Colonies able to grow at the non-permissive temperature (34°C) were selected. In total ∼2×10^7^ transformants were screened. Plasmids from ∼40 colonies were rescued in *Escherichia coli* and 17 different plasmids were identified as suppressors. Sixteen contained either *NHP6A* or *NHP6B*. One plasmid, pTK1385, contained the genomic sequence from 190959 to 198486 of chromosome XVI. Subcloning revealed that plasmid pTK1386 (pTK1385 digested with *Nhe*I and *Sac*I, blunt ended and re-ligated) containing the genomic sequence from 194878 to 198486 of chromosome XVI was a suppressor of *rsc8-ts16 nhp6ΔΔ* synthetic sickness. pTK1386 contained *YPL184c* as the only complete ORF. pTK1386 was digested with *Sna*BI and *Eco*RI and the 2357 bp fragment containing *YPL184c*, from 198277 to 195919 of the genomic sequence, was cloned into *Sma*I and *Eco*RI digested pRS423 (pTK839) resulting in plasmid pTK1395. pTK1395 was transformed into strain SG657 and was able to suppress its growth defect, and accordingly, we concluded that *2 µm-YPL184c* is a suppressor of the synthetic sickness of the *rsc8-ts16 nhp6ΔΔ* triple mutant.

### Construction of *MRN1-MYC*

The endogenous *MRN1-MYC* was constructed by inserting a Myc-Tag C-terminally on the *MRN1* gene by transformation and homologous recombination in yeast strain TG694 with two PCR fragments using *KANMX6* as the selection marker. DNA was amplified with oligonucleotides MYCa, sfh1d, sfh1e and MYCab using pTK1259 as the template. The manipulated region was subsequently sequenced to verify correct insertion.

*2 µm-MRN1* was Myc-tagged by inserting a Myc-tag C-terminally in the *MRN1* gene using *Sac*I-linearized plasmid pTK1395 and transformation and homologous recombination in yeast with a Myc-Tag containing PCR fragment. DNA was amplified with oligonucleotides MYCc and MYCab using pTK1259 as the template. Plasmid pTK1423 was rescued in *Escherichia coli* from His^+^ yeast transformants and sequenced to verify correct insertion. *2 µm-MRN1-MYC* suppresses the synthetic sickness of the *rsc8-ts16 nhp6ΔΔ* triple mutant (data not shown).

### Protein sequence analysis

Identifications and predictions based on the protein sequence of Mrn1 using the NCBI homepage searching for conserved domains (http://www.ncbi.nlm.nih.gov/Structure/cdd/wrpsb.cgi), the Robetta server [55] (http://robetta.bakerlab.org) and our own observations.

### Immuno blotting

Whole-cell extracts were prepared from 50 ml of cells growing exponentially in SC-His medium. Cells were collected and washed twice in lysis buffer (50 mM HEPES pH 7.5, 150 mM NaCl, 0.1 mM EDTA, 5 mM MgCl_2_, 0.5 mM dithiothreitiol (DTT), 0.25% NP-40) supplemented with Complete protease inhibitor cocktail (Roche). Cells were re-suspended in 400 µl lysis buffer and then lysed with glass beads (SIGMA) in a bead mill for 3×20 sec at 4°C. The cell debris was eliminated by centrifugation twice at 4°C (10×g, 5 min and 25 min, respectively). Protein concentration was measured with Bio-Rad Dc Protein Assay. Proteins were separated by SDS-PAGE and transferred to a mixed cellulose ester membrane and immuno-blotted with primary anti-Myc antibody (C3956-Sigma) or anti-HA antibody (12CA5 Roche). Proteins were visualized with anti-Rabbit or anti-Mouse Immuno-globulins/HRP (DAKO) and ECL Plus (GE Healthcare) with Hyperfilm ECL (GE Healthcare).

### Fluoroscence microscopy

Flouroscence microscopy was done with a Zeiss Imager Z1 using the channels for GFP, RFP and DIC. Logarithmically SC growing cells at 25°C or after a 30 min incubation at 37°C was harvested for microscopy. Strains SG1008 (*MRN1-GFP ADH1p-NLS-yEmRFP::URA3*) and SG1010 (*mex67-5 ADH1p-NLS-yEmRFP::URA3*) were constructed by integrating plasmid pML96-Int-ADH1p-NLS-yEmRFPrv-1 in the *ura3* locus in strains SG737 and SG736 (Table 2), respectively, after digestion with *Nsi*I. For quantification of each genotype and growth condition, 100–200 cells were inspected. *Error bars* indicate 95% confidence intervals.

### Northern protocol

RNA was electrophoresed in a 0.25 M formaldehyde agarose gel, transferred to a Hybond-NX (GE Healthcare) membrane by blotting overnight. RNA was cross-linked to the membrane in a Stratalinker (1200 µJ/cm^2^). Radioactively (^32^P) random primed labeled probes were produced with Prime-It® II Random Primer Labeling Kit (Stratagene) and purified with ProbeQuant G-25 Micro Colums (Amersham), utilizing gel purified PCR product as the template. The templates were produced with specific primers (Table 4) utilizing genomic yeast DNA as the template. Membranes were hybridized over night in a Hybaid oven at 42°C with Ultrahyb hybridization buffer (Ambion) and the membranes were washed as recommended by the manufacture. Hybridized probe were visualized and quantified using a Storm 840 Phosphorimager (Molecular Dynamics) and also visualized with Kodak BioMax MS Film when needed.

### Measurement of pre-mRNA accumulation by RT-qPCR

RNA was purified from exponentially growing cells with RNeasy Mini Kit (Qiagen). QuantiTect SYBR Green RT-PCR Kit (Qiagen) supplied with Fluorescein Calibration Dye (10 nM) (BIO-RAD) was used for the RT-qPCR amplifications done with the iCycler iQ (BIO-RAD). Data was analyzed with the iCycler iQ software (BIO-RAD). Standard deviations were calculated as suggested by Simon [56]. The sequence of used oligonucleotides is shown in Table 4.

### U4/U6 Assay

To visualize U4/U6 dimer, U4 Free and total U6 exponentially growing cells were harvested and resuspended in 250 µl of RNA extraction buffer (100 mM LiCl, 1 mM EDTA, 100 mM Tris-Cl (pH 7.5), 0.2% SDS) and transferred to a tube containing 250 µl glass beads and 250 µl Phenol-chloroform-isoamyl alchohol (25∶5∶0.2). Then the cells were lysed in a bead mill for 3×15 sec at 4°C. For non-denaturing gels the aqueous phase containing the RNA was mixed with one-third volume of loading dye (50% glycerol, 0.02% bromophenol blue) and loaded on a 6% non-denaturing polyacrylamide (29∶1) Tris-borate-EDTA gel containing 5% glycerol with 0.5 TBE as running buffer. The gel was run over night at 80 V at 4°. The gel was then soaked twice in 20 mM NaPO_4_ (pH 6.5), 8.3 M urea, 0.1% SDS at 37°, for 45 min and once in 20 mM NaPO_4_ (PH 6.5) at 4° for 1 hour. RNA was electrotransferred to a nylon membrane (Hybond-NX (GE Healthcare)) followed by UV cross-linking to the membrane in a Stratalinker (1200 µJ/cm^2^). For denaturing gels the aqueous phase was mixed with one volume of 2×RNA loading dye (Fermentas), denatured (70° for 10 min, on ice for 3 min) and loaded on a 6% denaturing polyacrylamide (29∶1) Tris-borate-EDTA gel containing 5% glycerol with 0.5×TBE as running buffer. Then the same protocol was used as for non-denaturing gels except the gel was only washed once in 20 mM NaPO_4_ (pH 6.5), 8.3 M urea, 0.1% SDS. Radioactively (^32^P) random primed labeled probes were produced with Prime-It® II Random Primer Labeling Kit (Stratagene) and purified with ProbeQuant G-25 Micro Colums (Amersham) with single stranded oligonucleotides Snr14 or Snr6a as templates (Table 4). The template for the U1 probe was generated by PCR using genomic yeast DNA and primers Snr19a and Snr19b (Table 4). Hybridization was at 42° with Rapid-Hyb buffer (GE Healthcare) in a Hybaid oven over night followed by 2×5 min washes in 6×SSC, 0.2% SDS and one 15 min wash in 2×SSC, 0.2% SDS at 42°. Hybridized probe were visualized and quantified using a Storm 840 Phosphorimager (Molecular Dynamics) and also visualized with Kodak BioMax MS Film when needed. This protocol was modified from Lygerou et al. [57].

## Supporting Information

Figure S1 Schematic representation of *RPS11B*, *ASC1*, *ACT1*, and *ECM33* probes and qPCR primers. The relative position of the DNA fragments used as *RPS11B* and *ECM33* Northern probes as well as the relative position of the primers used for the qPCR analyses are depicted.
(TIF)

Figure S2 *rsc1Δ nhp6ΔΔ* and *snt309Δ* cells accumulate unspliced transcripts. Total RNA isolated from logarithmically SC-His growing cells at 25°C or after a 2 hour shift at 37°C amplified by RT-qPCR with *ECM33-*, *ACT1-*, *ASC1-* or *RPS11B-*specific primers. The ratio intron-3′exon junction RT-PCR-amplificate/3′exon RT-PCR-amplificate. The ratio in wild type cells at 25°C was arbitrarily set to 1. ND: Not determined. Wild type: SG632; *rsc1Δ nhp6ΔΔ*: SG518; *snt309Δ*: SG648.
(TIF)

Figure S3 *rsc1Δ* and *rsc8-ts16* or *nhp6ΔΔ* mutants do not generally accumulate unspliced mRNA at 37°C. Total RNA isolated from logarithmically SC-His growing cells at 25°C or after a 2 hour shift at 37°C amplified by RT-qPCR with *ECM33-*, *ACT1-*, *ASC1-* or *RPS11B-*specific primers. The ratio intron-3′exon junction RT-PCR-amplificate/3′exon RT-PCR-amplificate. The ratio in wild type cells at 25°C was arbitrarily set to 1. Wild type: SG632; *rsc1Δ*: SG416, *rsc8-ts16*: SG360 and *nhp6ΔΔ*: SG306.
(TIF)

## References

[1] Workman JL (2006) Nucleosome displacement in transcription. Genes Dev 20: 2009–2017. 16882978 10.1101/gad.1435706

[2] Becker PB, Horz W (2002) ATP-dependent nucleosome remodeling. Annu Rev Biochem 71: 247–273. 12045097 10.1146/annurev.biochem.71.110601.135400

[3] Moreira JM, Holmberg S (1999) Transcriptional repression of the yeast CHA1 gene requires the chromatin-remodeling complex RSC. Embo J 18: 2836–2844. 10329629 10.1093/emboj/18.10.2836 PMC1171364

[4] Angus-Hill ML, Schlichter A, Roberts D, Erdjument-Bromage H, Tempst P, et al (2001) A Rsc3/Rsc30 zinc cluster dimer reveals novel roles for the chromatin remodeler RSC in gene expression and cell cycle control. Mol Cell 7: 741–751. 11336698 10.1016/s1097-2765(01)00219-2

[5] Chai B, Huang J, Cairns BR, Laurent BC (2005) Distinct roles for the RSC and Swi/Snf ATP-dependent chromatin remodelers in DNA double-strand break repair. Genes Dev 19: 1656–1661. 16024655 10.1101/gad.1273105 PMC1176001

[6] Hsu JM, Huang J, Meluh PB, Laurent BC (2003) The yeast RSC chromatin-remodeling complex is required for kinetochore function in chromosome segregation. Mol Cell Biol 23: 3202–3215. 12697820 10.1128/MCB.23.9.3202-3215.2003 PMC153182

[7] Ng HH, Robert F, Young RA, Struhl K (2002) Genome-wide location and regulated recruitment of the RSC nucleosome-remodeling complex. Genes Dev 16: 806–819. 11937489 10.1101/gad.978902 PMC186327

[8] Parnell TJ, Huff JT, Cairns BR (2008) RSC regulates nucleosome positioning at Pol II genes and density at Pol III genes. Embo J 27: 100–110. 18059476 10.1038/sj.emboj.7601946 PMC2206128

[9] Bustin M (1999) Regulation of DNA-dependent activities by the functional motifs of the high-mobility-group chromosomal proteins. Mol Cell Biol 19: 5237–5246. 10409715 10.1128/mcb.19.8.5237 PMC84367

[10] Costigan C, Kolodrubetz D, Snyder M (1994) NHP6A and NHP6B, which encode HMG1-like proteins, are candidates for downstream components of the yeast SLT2 mitogen-activated protein kinase pathway. Mol Cell Biol 14: 2391–2403. 8139543 10.1128/mcb.14.4.2391-2403.1994 PMC358606

[11] Moreira JM, Holmberg S (2000) Chromatin-mediated transcriptional regulation by the yeast architectural factors NHP6A and NHP6B. Embo J 19: 6804–6813. 11118215 10.1093/emboj/19.24.6804 PMC305882

[12] Brewster NK, Johnston GC, Singer RA (2001) A bipartite yeast SSRP1 analog comprised of Pob3 and Nhp6 proteins modulates transcription. Mol Cell Biol 21: 3491–3502. 11313475 10.1128/MCB.21.10.3491-3502.2001 PMC100271

[13] Lopez S, Livingstone-Zatchej M, Jourdain S, Thoma F, Sentenac A, et al (2001) High-mobility-group proteins NHP6A and NHP6B participate in activation of the RNA polymerase III SNR6 gene. Mol Cell Biol 21: 3096–3104. 11287614 10.1128/MCB.21.9.3096-3104.2001 PMC86937

[14] Kruppa M, Moir RD, Kolodrubetz D, Willis IM (2001) Nhp6, an HMG1 protein, functions in SNR6 transcription by RNA polymerase III in S. cerevisiae. Mol Cell 7: 309–318. 11239460 10.1016/s1097-2765(01)00179-4

[15] Wang W, Chi T, Xue Y, Zhou S, Kuo A, et al (1998) Architectural DNA binding by a high-mobility-group/kinesin-like subunit in mammalian SWI/SNF-related complexes. Proc Natl Acad Sci U S A 95: 492–498. 9435219 10.1073/pnas.95.2.492 PMC18447

[16] Papoulas O, Daubresse G, Armstrong JA, Jin J, Scott MP, et al (2001) The HMG-domain protein BAP111 is important for the function of the BRM chromatin-remodeling complex in vivo. Proc Natl Acad Sci U S A 98: 5728–5733. 11331758 10.1073/pnas.091533398 PMC33281

[17] Szerlong H, Saha A, Cairns BR (2003) The nuclear actin-related proteins Arp7 and Arp9: a dimeric module that cooperates with architectural proteins for chromatin remodeling. Embo J 22: 3175–3187. 12805231 10.1093/emboj/cdg296 PMC162148

[18] Morillon A, Karabetsou N, O'Sullivan J, Kent N, Proudfoot N, et al (2003) Isw1 chromatin remodeling ATPase coordinates transcription elongation and termination by RNA polymerase II. Cell 115: 425–435. 14622597 10.1016/s0092-8674(03)00880-8

[19] Tyagi A, Ryme J, Brodin D, Ostlund Farrants AK, Visa N (2009) SWI/SNF associates with nascent pre-mRNPs and regulates alternative pre-mRNA processing. PLoS Genet 5: e1000470. 19424417 10.1371/journal.pgen.1000470 PMC2669885

[20] Batsche E, Yaniv M, Muchardt C (2006) The human SWI/SNF subunit Brm is a regulator of alternative splicing. Nat Struct Mol Biol 13: 22–29. 16341228 10.1038/nsmb1030

[21] Allemand E, Batsche E, Muchardt C (2008) Splicing, transcription, and chromatin: a menage a trois. Curr Opin Genet Dev 18: 145–151. 18372167 10.1016/j.gde.2008.01.006

[22] Neugebauer KM (2002) On the importance of being co-transcriptional. J Cell Sci 115: 3865–3871. 12244124 10.1242/jcs.00073

[23] Moore MJ, Schwartzfarb EM, Silver PA, Yu MC (2006) Differential recruitment of the splicing machinery during transcription predicts genome-wide patterns of mRNA splicing. Mol Cell 24: 903–915. 17189192 10.1016/j.molcel.2006.12.006

[24] Tardiff DF, Lacadie SA, Rosbash M (2006) A genome-wide analysis indicates that yeast pre-mRNA splicing is predominantly posttranscriptional. Mol Cell 24: 917–929. 17189193 10.1016/j.molcel.2006.12.002 PMC1828117

[25] Jurica MS, Moore MJ (2003) Pre-mRNA splicing: awash in a sea of proteins. Mol Cell 12: 5–14. 12887888 10.1016/s1097-2765(03)00270-3

[26] Nilsen TW (2003) The spliceosome: the most complex macromolecular machine in the cell? Bioessays 25: 1147–1149. 14635248 10.1002/bies.10394

[27] Dellaire G, Makarov EM, Cowger JJ, Longman D, Sutherland HG, et al (2002) Mammalian PRP4 kinase copurifies and interacts with components of both the U5 snRNP and the N-CoR deacetylase complexes. Mol Cell Biol 22: 5141–5156. 12077342 10.1128/MCB.22.14.5141-5156.2002 PMC139773

[28] Chen YI, Moore RE, Ge HY, Young MK, Lee TD, et al (2007) Proteomic analysis of in vivo-assembled pre-mRNA splicing complexes expands the catalog of participating factors. Nucleic Acids Res 35: 3928–3944. 17537823 10.1093/nar/gkm347 PMC1919476

[29] Chan SP, Cheng SC (2005) The Prp19-associated complex is required for specifying interactions of U5 and U6 with pre-mRNA during spliceosome activation. J Biol Chem 280: 31190–31199. 15994330 10.1074/jbc.M505060200

[30] Chan SP, Kao DI, Tsai WY, Cheng SC (2003) The Prp19p-associated complex in spliceosome activation. Science 302: 279–282. 12970570 10.1126/science.1086602

[31] Carlson M, Botstein D (1982) Two differentially regulated mRNAs with different 5′ ends encode secreted with intracellular forms of yeast invertase. Cell 28: 145–154. 7039847 10.1016/0092-8674(82)90384-1

[32] Wong MC, Scott-Drew SR, Hayes MJ, Howard PJ, Murray JA (2002) RSC2, encoding a component of the RSC nucleosome remodeling complex, is essential for 2 microm plasmid maintenance in Saccharomyces cerevisiae. Mol Cell Biol 22: 4218–4229. 12024034 10.1128/MCB.22.12.4218-4229.2002 PMC133863

[33] Maris C, Dominguez C, Allain FH (2005) The RNA recognition motif, a plastic RNA-binding platform to regulate post-transcriptional gene expression. Febs J 272: 2118–2131. 15853797 10.1111/j.1742-4658.2005.04653.x

[34] Huh WK, Falvo JV, Gerke LC, Carroll AS, Howson RW, et al (2003) Global analysis of protein localization in budding yeast. Nature 425: 686–691. 14562095 10.1038/nature02026

[35] Segref A, Sharma K, Doye V, Hellwig A, Huber J, et al (1997) Mex67p, a novel factor for nuclear mRNA export, binds to both poly(A)+ RNA and nuclear pores. Embo J 16: 3256–3271. 9214641 10.1093/emboj/16.11.3256 PMC1169942

[36] Chen HR, Jan SP, Tsao TY, Sheu YJ, Banroques J, et al (1998) Snt309p, a component of the Prp19p-associated complex that interacts with Prp19p and associates with the spliceosome simultaneously with or immediately after dissociation of U4 in the same manner as Prp19p. Mol Cell Biol 18: 2196–2204. 9528791 10.1128/mcb.18.4.2196 PMC121462

[37] Costanzo M, Baryshnikova A, Bellay J, Kim Y, Spear ED, et al (2010) The genetic landscape of a cell. Science 327: 425–431. 20093466 10.1126/science.1180823 PMC5600254

[38] Wilson B, Erdjument-Bromage H, Tempst P, Cairns BR (2006) The RSC chromatin remodeling complex bears an essential fungal-specific protein module with broad functional roles. Genetics 172: 795–809. 16204215 10.1534/genetics.105.047589 PMC1456245

[39] Raghunathan PL, Guthrie C (1998) A spliceosomal recycling factor that reanneals U4 and U6 small nuclear ribonucleoprotein particles. Science 279: 857–860. 9452384 10.1126/science.279.5352.857

[40] Blanton S, Srinivasan A, Rymond BC (1992) PRP38 encodes a yeast protein required for pre-mRNA splicing and maintenance of stable U6 small nuclear RNA levels. Mol Cell Biol 12: 3939–3947. 1508195 10.1128/mcb.12.9.3939-3947.1992 PMC360275

[41] Mayes AE, Verdone L, Legrain P, Beggs JD (1999) Characterization of Sm-like proteins in yeast and their association with U6 snRNA. Embo J 18: 4321–4331. 10428970 10.1093/emboj/18.15.4321 PMC1171508

[42] Xie J, Beickman K, Otte E, Rymond BC (1998) Progression through the spliceosome cycle requires Prp38p function for U4/U6 snRNA dissociation. Embo J 17: 2938–2946. 9582287 10.1093/emboj/17.10.2938 PMC1170634

[43] Rymond BC (1993) Convergent transcripts of the yeast PRP38-SMD1 locus encode two essential splicing factors, including the D1 core polypeptide of small nuclear ribonucleoprotein particles. Proc Natl Acad Sci U S A 90: 848–852. 8430095 10.1073/pnas.90.3.848 PMC45767

[44] Hu J, Xu Y, Schappert K, Harrington T, Wang A, et al (1994) Mutational analysis of the PRP4 protein of Saccharomyces cerevisiae suggests domain structure and snRNP interactions. Nucleic Acids Res 22: 1724–1734. 8202378 10.1093/nar/22.9.1724 PMC308056

[45] Cooper M, Johnston LH, Beggs JD (1995) Identification and characterization of Uss1p (Sdb23p): a novel U6 snRNA-associated protein with significant similarity to core proteins of small nuclear ribonucleoproteins. Embo J 14: 2066–2075. 7744012 10.1002/j.1460-2075.1995.tb07198.x PMC398307

[46] Anthony JG, Weidenhammer EM, Woolford JL (1997) The yeast Prp3 protein is a U4/U6 snRNP protein necessary for integrity of the U4/U6 snRNP and the U4/U6.U5 tri-snRNP. Rna 3: 1143–1152. 9326489 PMC1369556

[47] Chen CH, Kao DI, Chan SP, Kao TC, Lin JY, et al (2006) Functional links between the Prp19-associated complex, U4/U6 biogenesis, and spliceosome recycling. Rna 12: 765–774. 16540691 10.1261/rna.2292106 PMC1440898

[48] Soutourina J, Bordas-Le Floch V, Gendrel G, Flores A, Ducrot C, et al (2006) Rsc4 connects the chromatin remodeler RSC to RNA polymerases. Mol Cell Biol 26: 4920–4933. 16782880 10.1128/MCB.00415-06 PMC1489167

[49] Herrick D, Parker R, Jacobson A (1990) Identification and comparison of stable and unstable mRNAs in Saccharomyces cerevisiae. Mol Cell Biol 10: 2269–2284. 2183028 10.1128/mcb.10.5.2269-2284.1990 PMC360574

[50] Jimenez A, Tipper DJ, Davies J (1973) Mode of action of thiolutin, an inhibitor of macromolecular synthesis in Saccharomyces cerevisiae. Antimicrob Agents Chemother 3: 729–738. 4597739 10.1128/aac.3.6.729 PMC444489

[51] Moenne A, Camier S, Anderson G, Margottin F, Beggs J, et al (1990) The U6 gene of Saccharomyces cerevisiae is transcribed by RNA polymerase C (III) in vivo and in vitro. Embo J 9: 271–277. 2403927 10.1002/j.1460-2075.1990.tb08105.x PMC551658

[52] Hogan DJ, Riordan DP, Gerber AP, Herschlag D, Brown PO (2008) Diverse RNA-Binding Proteins Interact with Functionally Related Sets of RNAs, Suggesting an Extensive Regulatory System. PLoS Biol 6: e255. 18959479 10.1371/journal.pbio.0060255 PMC2573929

[53] Sherman F (1991) Getting started with yeast. Methods Enzymol 194: 3–21. 2005794 10.1016/0076-6879(91)94004-v

[54] Ito H, Fukuda Y, Murata K, Kimura A (1983) Transformation of intact yeast cells treated with alkali cations. J Bacteriol 153: 163–168. 6336730 10.1128/jb.153.1.163-168.1983 PMC217353

[55] Kim DE, Chivian D, Baker D (2004) Protein structure prediction and analysis using the Robetta server. Nucleic Acids Res 32: W526–531. 15215442 10.1093/nar/gkh468 PMC441606

[56] Simon P (2003) Q-Gene: processing quantitative real-time RT-PCR data. Bioinformatics 19: 1439–1440. 12874059 10.1093/bioinformatics/btg157

[57] Lygerou Z, Christophides G, Seraphin B (1999) A novel genetic screen for snRNP assembly factors in yeast identifies a conserved protein, Sad1p, also required for pre-mRNA splicing. Mol Cell Biol 19: 2008–2020. 10022888 10.1128/mcb.19.3.2008 PMC83994

[58] Paull TT, Carey M, Johnson RC (1996) Yeast HMG proteins NHP6A/B potentiate promoter-specific transcriptional activation in vivo and assembly of preinitiation complexes in vitro. Genes Dev 10: 2769–2781. 8946917 10.1101/gad.10.21.2769

[59] Du J, Nasir I, Benton BK, Kladde MP, Laurent BC (1998) Sth1p, a Saccharomyces cerevisiae Snf2p/Swi2p homolog, is an essential ATPase in RSC and differs from Snf/Swi in its interactions with histones and chromatin-associated proteins. Genetics 150: 987–1005. 9799253 10.1093/genetics/150.3.987 PMC1460405

[60] Treich I, Ho L, Carlson M (1998) Direct interaction between Rsc6 and Rsc8/Swh3,two proteins that are conserved in SWI/SNF-related complexes. Nucleic Acids Res 26: 3739–3745. 9685490 10.1093/nar/26.16.3739 PMC147781

[61] Vijayraghavan U, Company M, Abelson J (1989) Isolation and characterization of pre-mRNA splicing mutants of Saccharomyces cerevisiae. Genes Dev 3: 1206–1216. 2676722 10.1101/gad.3.8.1206

[62] Cao Y, Cairns BR, Kornberg RD, Laurent BC (1997) Sfh1p, a component of a novel chromatin-remodeling complex, is required for cell cycle progression. Mol Cell Biol 17: 3323–3334. 9154831 10.1128/mcb.17.6.3323 PMC232185

[63] Rose M, Grisafi P, Botstein D (1984) Structure and function of the yeast URA3 gene: expression in Escherichia coli. Gene 29: 113–124. 6092217 10.1016/0378-1119(84)90172-0

[64] Sikorski RS, Hieter P (1989) A system of shuttle vectors and yeast host strains designed for efficient manipulation of DNA in Saccharomyces cerevisiae. Genetics 122: 19–27. 2659436 10.1093/genetics/122.1.19 PMC1203683

[65] Bahler J, Wu JQ, Longtine MS, Shah NG, McKenzie A III, et al (1998) Heterologous modules for efficient and versatile PCR-based gene targeting in Schizosaccharomyces pombe. Yeast 14: 943–951. 9717240 10.1002/(SICI)1097-0061(199807)14:10<943::AID-YEA292>3.0.CO;2-Y

