# Supplementary figures and images for: MRN1 Implicates Chromatin Remodeling Complexes and Architectural Factors in mRNA Maturation
Source: PLoS One. 2012 Sep 18;7(9):e44373. doi: 10.1371/journal.pone.0044373 (PMC3445587; doi:10.1371/journal.pone.0044373)

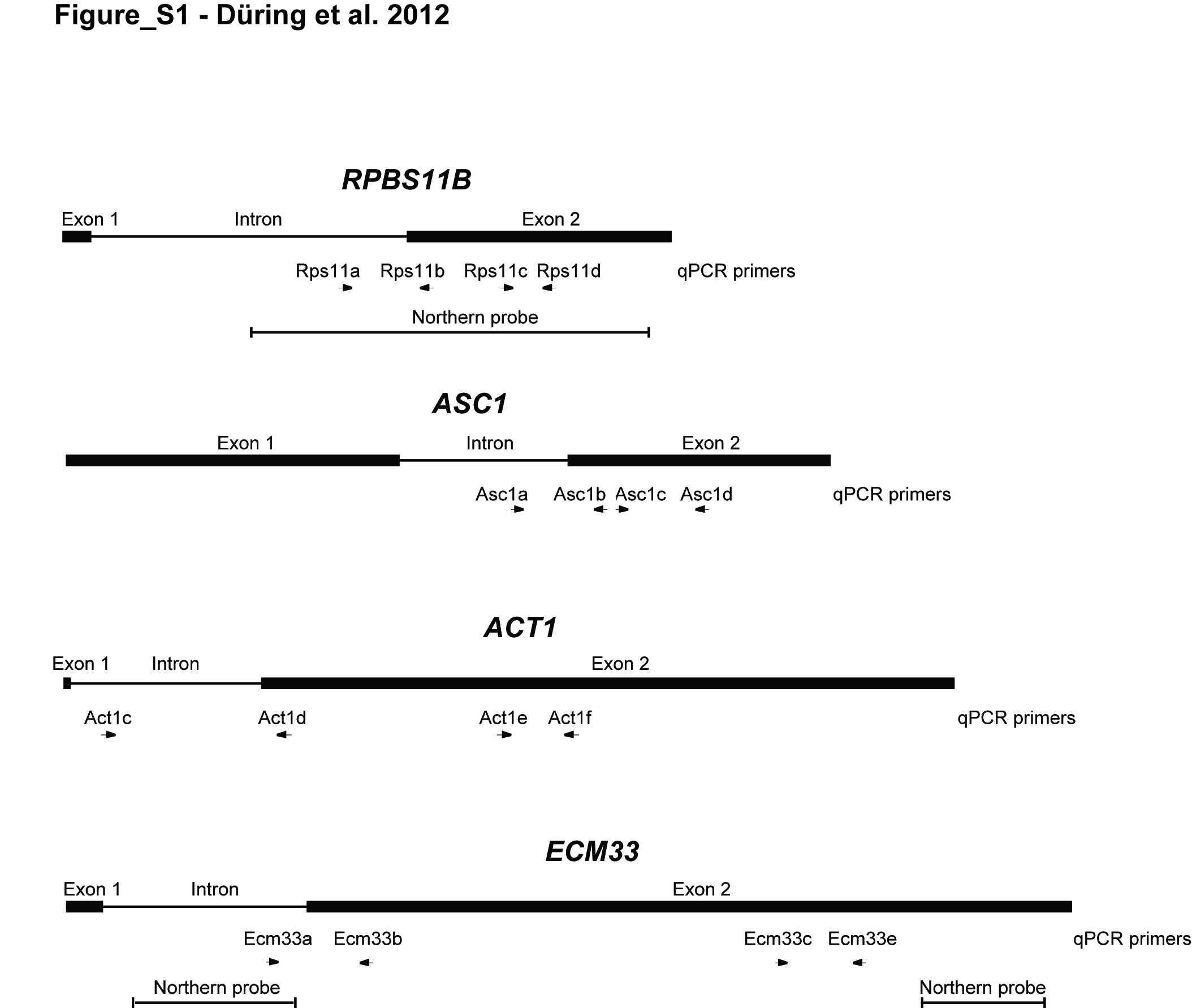

Supplement: Figure S1 — Schematic representation of RPS11B, ASC1, ACT1, and ECM33 probes and qPCR primers. The relative position of the DNA fragments used as RPS11B and ECM33 Northern probes as well as the relative position of the primers used for the qPCR analyses are depicted. (TIF) [file pone.0044373.s001.tif]

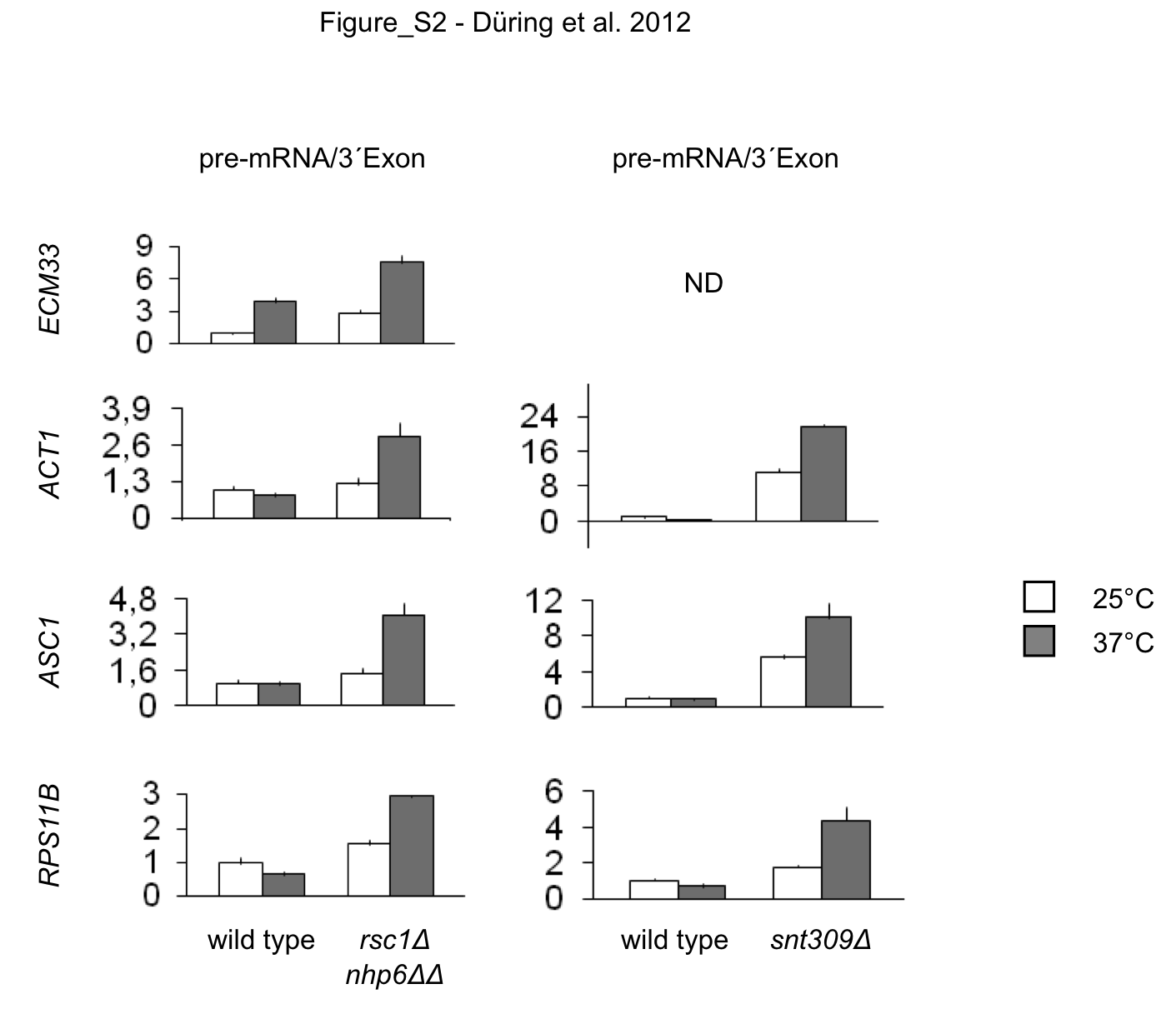

Supplement: Figure S2 — rsc1Δ nhp6ΔΔ and snt309Δ cells accumulate unspliced transcripts. Total RNA isolated from logarithmically SC-His growing cells at 25°C or after a 2 hour shift at 37°C amplified by RT-qPCR with ECM33-, ACT1-, ASC1- or RPS11B-specific primers. The ratio intron-3′exon junction RT-PCR-amplificate/3′exon RT-PCR-amplificate. The ratio in wild type cells at 25°C was arbitrarily set to 1. ND: Not determined. Wild type: SG632; rsc1Δ nhp6ΔΔ: SG518; snt309Δ: SG648. (TIF) [file pone.0044373.s002.tif]

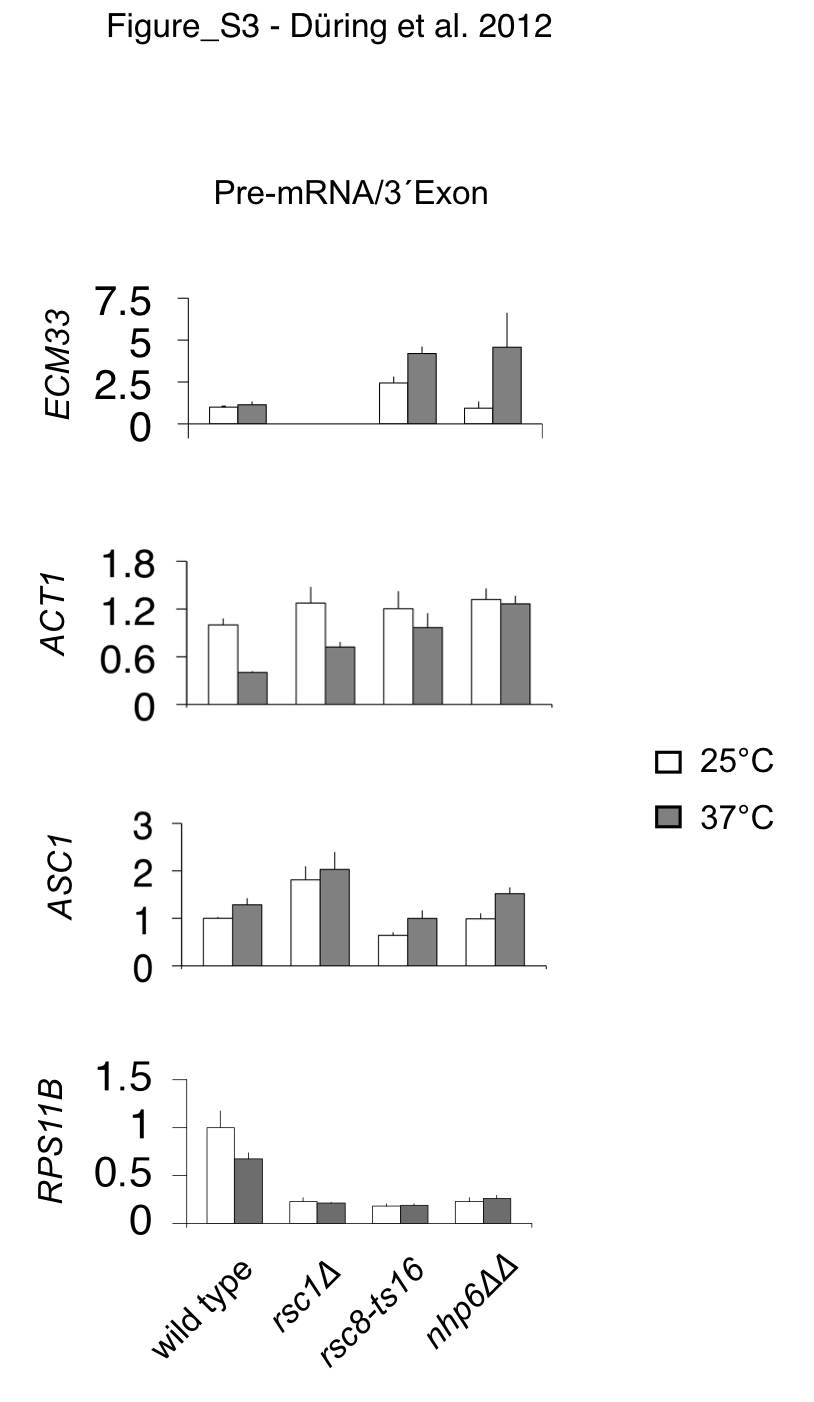

Supplement: Figure S3 — rsc1Δ and rsc8-ts16 or nhp6ΔΔ mutants do not generally accumulate unspliced mRNA at 37°C. Total RNA isolated from logarithmically SC-His growing cells at 25°C or after a 2 hour shift at 37°C amplified by RT-qPCR with ECM33-, ACT1-, ASC1- or RPS11B-specific primers. The ratio intron-3′exon junction RT-PCR-amplificate/3′exon RT-PCR-amplificate. The ratio in wild type cells at 25°C was arbitrarily set to 1. Wild type: SG632; rsc1Δ: SG416, rsc8-ts16: SG360 and nhp6ΔΔ: SG306. (TIF) [file pone.0044373.s003.tif]
